# Species-specific barriers restrict virus spillover potential across the *Caenorhabditis* genus

**DOI:** 10.1371/journal.pbio.3003965

**Published:** 2026-08-18

**Authors:** Dominik Herek, Juan C. Muñoz-Sánchez, Ana Villena-Giménez, Victoria G. Castiglioni, Santiago F. Elena

**Affiliations:** 1 Instituto de Biología Integrativa de Sistemas, I^2^SysBio, CSIC-Universitat de València, Paterna, València, Spain; 2 Department de Física Teòrica, Universitat de València, Burjassot, València, Spain; 3 Department of Molecular Genetics, University of Toronto, Toronto, Ontario, Canada; 4 The Santa Fe Institute, Santa Fe, New Mexico, United States of America; ETH Zurich Department of Environmental Systems Science: Eidgenossische Technische Hochschule Departement Umweltsystemwissenschaften, SWITZERLAND

## Abstract

Host range expansion and virus emergence depend on whether viral life-cycle processes align with the ecological, developmental and physiological traits of new hosts. Spillover therefore succeeds only when pathogens clear a hierarchy of mechanistic barriers, from entry and replication to transmission and evolutionary persistence, but how these barriers map onto whole-organism host competence remains poorly resolved. Here we dissect spillover barriers experimentally using the *Caenorhabditis*-Orsay virus system, integrating within-host viral kinetics, cellular progression, transmission, virulence and experimental evolution across six closely related host species. We show that host species identity deterministically reshapes the timing and completeness of the viral life cycle, generating distinct host-competence phenotypes that range from permissive to restrictive and evolutionary dead-end hosts. Alternative hosts disrupt viral life-cycle synchrony through delayed replication, imbalanced genomic segment production, impaired egress or truncated infection windows, reducing transmission despite occasional high viral loads. These mechanistic mismatches prevent sustained viral adaptation upon serial passage, revealing how multiple partially permeable barriers compound to block emergence. By resolving spillover barriers across biological scales, our results provide a mechanistic framework linking viral life-history traits to eco-evolutionary theory of host range, and show how temporal and stoichiometric mismatches can determine whether cross-species infections become epidemiologically and evolutionarily viable.

## Abstract 2

Why do most cross-species virus jumps fail? Using Orsay virus across multiple *Caenorhabditis* species, this study reveals how mismatches in replication, genome balance, and transmission create layered barriers, showing that viral load alone does not predict emergence or evolutionary success.

## Introduction

Host range expansion is the critical first step in the emergence of viral diseases, yet successful spillover remains rare despite frequent cross-species exposure. Ecological contact alone is insufficient: pathogens must sequentially overcome a hierarchy of mechanistic barriers spanning cellular infection, within-host amplification, transmission, and evolutionary persistence in the new host [1,2]. Spillover theory therefore predicts that emergence depends not solely on molecular compatibility, but on the alignment of viral life-cycle processes with host developmental schedules, immune dynamics and ecological context [1,3,4]. When this alignment fails, because replication is mistimed, genomes are imbalanced, or virions are not effectively shed, infections stall, persist as dead ends, or collapse before adaptation can occur.

Pandemics underscore the consequences of this process [5,6]. Many emerge from zoonotic jumps, particularly by RNA viruses, yet sustained spread after cross-species contact is uncommon because host defenses impose strong barriers to establishment and transmission [1,7–13]. Spillover therefore occurs more frequently between closely related hosts, where physiological and ecological mismatches are reduced. RNA viruses are nevertheless well positioned to explore novel hosts given their short generation times, large population sizes, and high mutation rates, which accelerate exploration of adaptive space [2,14,15]. Understanding how viral life cycles interact with host traits across related species is thus essential to anticipate host range and emergence potential.

Despite the centrality of this barrier-based view, empirical tests remain limited. Most systems measure only single components of host competence, such as infection prevalence or viral load, yet competence is inherently multicomponent. Hosts that permit entry may fail at replication; hosts that sustain replication may block transmission; and hosts that transmit efficiently may still function as evolutionary sinks. Resolving how these barriers combine within real organisms requires tractable systems that allow direct, multiscale measurement of viral progression under standardized conditions.

Most prior work addressing spillover relies on retrospective analyses or diversity surveys that are invaluable for surveillance but limited for hypothesis testing [16,17]. Controlled experimental models offer a complementary route: phage–bacteria systems and cell culture have produced key insights but lack whole-organism ecological and developmental context [18–24]. Plant virus models have successfully incorporated organismal context and evolutionary dynamics [25–27], yet remain distant from animal host biology. Mammalian models can explicitly link viral load to transmission, but ethical and logistical constraints restrict scale and replication [28–30]. Together, these gaps motivate the need for experimentally tractable animal systems that resolve spillover-relevant traits (including replication timing, tissue tropism, immune clearance, shedding, and transmission) across closely related hosts.

The *Caenorhabditis*–Orsay virus (OrV) pathosystem provides such a model for mechanistic dissection of spillover barriers. *Caenorhabditis elegans* is a free-living bacterivore with well-characterized ecology and exceptional genetic tractability [31], widely used to study development [32], aging [33], and innate immunity [34]. The discovery of OrV as a natural pathogen of *C. elegans* [35,36] enabled whole-organism analyses of viral infection, antiviral defenses and experimental evolution [37–41]. Combined with extensive natural diversity across the genus, this system provides an opportunity to test eco-evolutionary predictions of host range and spillover under controlled conditions [42,43].

OrV is a positive-sense, single-stranded RNA virus with a bipartite genome. RNA1 encodes the RNA-dependent RNA polymerase, while RNA2 encodes the capsid protein, the δ protein required for nonlytic egress, and a capsid-δ fusion involved in entry [35,44–46]. Infections are typically confined to one or a few intestinal cells [39] and are countered in *C. elegans* by RNA interference, RNA uridylation and the intracellular pathogen response [47–49]. Fitness effects are modest, including small developmental delays and reductions in body size, fertility and life span [41,47,50,51]. Across host genotypes and environments, OrV displays a conserved early replication peak around 12 hours post-inoculation (hpi), whereas later infection dynamics vary widely, implicating both viral and host factors in shaping infection outcomes [39,41,50,51].

The *Caenorhabditis* genus comprises >65 species with substantial genetic and ecological diversity [52,53]. Several species are susceptible to OrV, but susceptibility and infection outcomes vary widely, from permissive to strongly restrictive species [42]. While prior work has emphasized predicting establishment using infection intensity, prevalence and shedding [43], we still lack a mechanistic, multiscale understanding of how viral life cycles succeed or fail across hosts.

Here, we apply a unified experimental framework to six *Caenorhabditis* species, integrating quantitative viral kinetics, single-animal heterogeneity, cellular localization, transmission assays, virulence measurements, and experimental evolution. We show that host species impose distinct temporal and mechanistic constraints on the viral life cycle, shaping susceptibility, replication efficiency, genome stoichiometry, egress, transmission probability, and evolutionary persistence. Framed within spillover theory, our results resolve host-competence phenotypes and demonstrate how life-cycle mismatches generate eco-evolutionary barriers to emergence, linking organismal infection dynamics to general principles of host range evolution [1,54–60].

## Results

**Fig 1:**
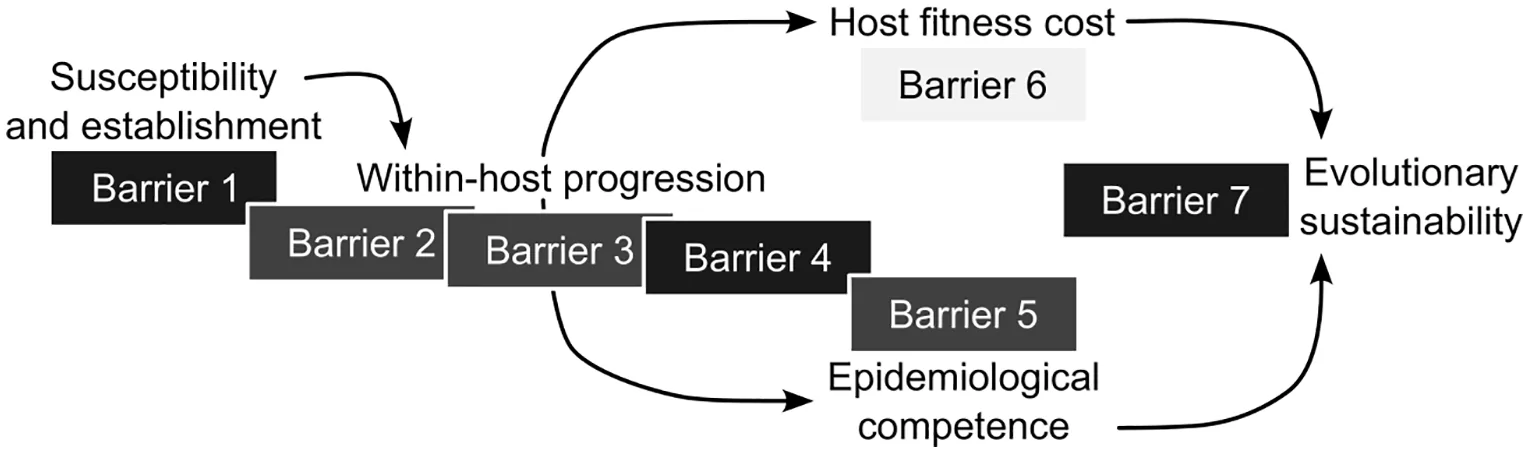
Barrier-resolved framework for OrV host competence across *Caenorhabditis* species. Conceptual summary of the seven barriers evaluated in this study and the expected empirical signature if each barrier limits OrV host competence. The framework distinguishes early susceptibility/establishment, within-host replication kinetics, RNA2:RNA1 genome-segment balance, luminal localization consistent with egress or viral material accumulation, functional transmission, virulence, and evolutionary persistence under serial passage.

To determine why OrV spillover succeeds in some *Caenorhabditis* hosts but fails in others, we organized the Results around a barrier-resolved model of host competence (Fig 1). In this framework, exposure alone is insufficient for emergence: OrV must pass through a series of mechanistic filters that include susceptibility and early-cycle progression, within-host replication, balanced accumulation of RNA1 and RNA2, progression toward egress or luminal localization, functional transmission, measurable host fitness effects, and evolutionary maintenance during serial passage (Fig 1). Each barrier is partially permeable, and failure at any one step may reduce host competence even when earlier steps appear permissive. We therefore evaluated OrV infection across six host species, *C. elegans*, *Caenorhabditis tropicalis*, *Caenorhabditis wallacei*, *Caenorhabditis remanei*, *Caenorhabditis macrosperma*, and *Caenorhabditis sulstoni*, using complementary assays that map onto these successive barriers. *C. elegans*, *C. tropicalis*, *C. wallacei*, and *C. remanei* belong to the *Elegans* group, whereas *C. macrosperma* and *C. sulstoni* belong to the *Japonica* group [53]. We begin with the first barrier, susceptibility and early-cycle progression, measured by smiFISH detection of OrV RNA after inoculation.

### Barrier 1: Susceptibility, establishment and early-cycle progression

To examine host susceptibility and early infection outcomes in the selected species [42], we performed single-molecule fluorescence in situ hybridization (smiFISH) targeting OrV positive-strand RNA. Synchronized animals inoculated at hatching were collected at 24 hpi for all species and additionally at 14 hpi for *C. elegans* and *C. remanei*, and the proportion of animals with a positive smiFISH signal was determined. Because this assay detects viral RNA after inoculation, smiFISH positivity does not by itself distinguish among viral entry, uncoating, initiation of replication, persistence of incoming viral RNA, or rapid clearance. Thus, we interpret these data as measuring detectable viral RNA associated with susceptibility, establishment, and early-cycle progression, rather than as direct evidence for viral entry alone.

**Fig 2:**
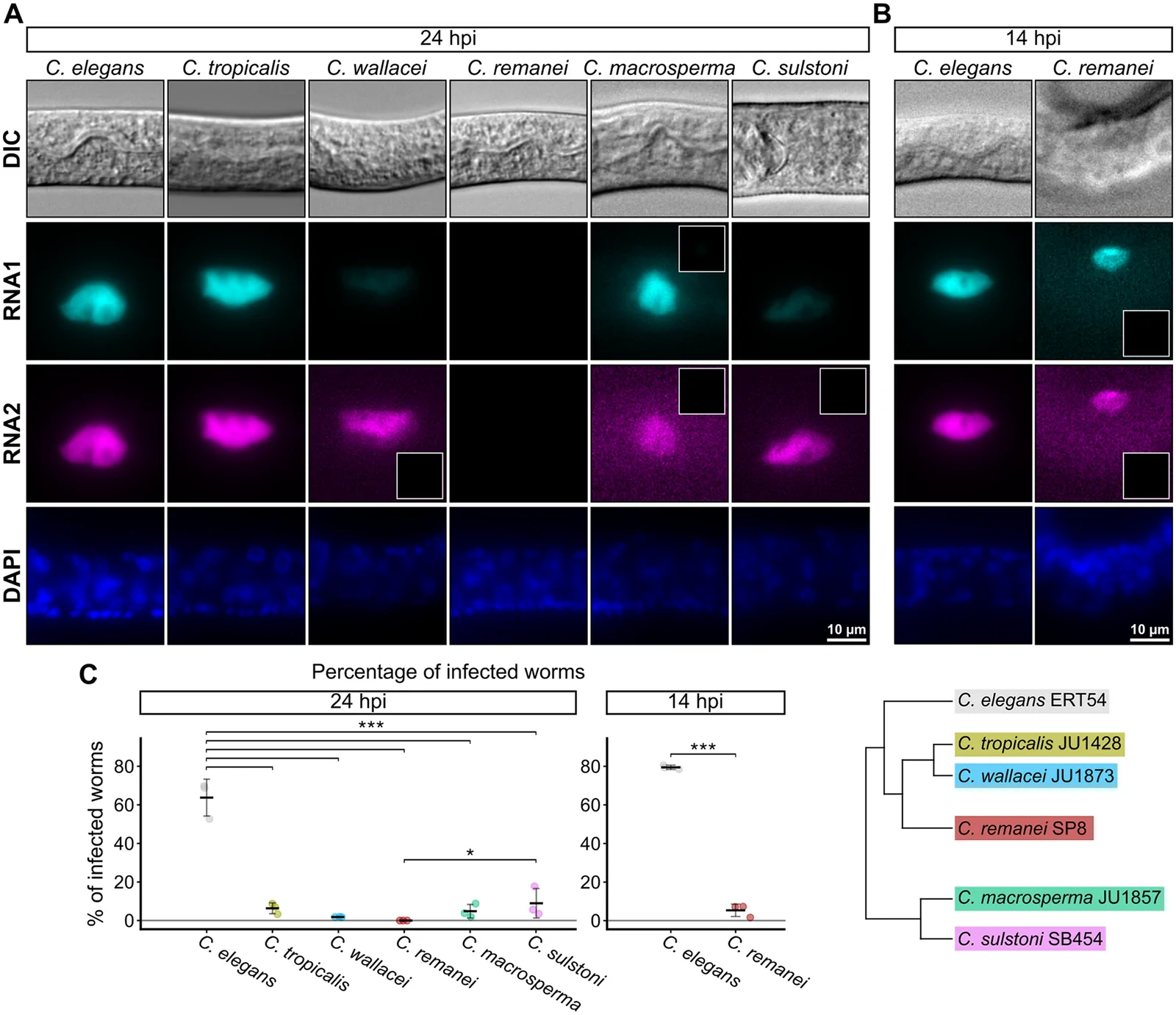
Representative smiFISH images of OrV infection in alternative host species. **(A)** *C. elegans* and the alternative host species at 24 hours post-inoculation (hpi). **(B)** *C. elegans* and *C. remanei* at 14 hpi. The brightness levels of all images were equalized to allow for comparisons. All images are displayed with the same brightness and contrast levels to allow for visual comparisons, except for the images that have insets. The inset displays same brightness and contrast as the other images, and the magnified images have increased brightness to visualize low signal. Infection was not detected in any *C. remanei* worms at 24 hpi. Top: differential interference contrast (DIC), OrV RNA1 (cyan), OrV RNA2 (magenta), DAPI (blue). **(C)** Percentage of infected worms at 24 and 14 hpi determined by counting the number of animals with a positive smiFISH signal (*n* = 3 replicates of 56 ± 4 animals). The legend showing colors used for each species also shows phylogenetic relationships adapted from Stevens and colleagues (2020). The data underlying this Figure can be found in https://doi.org/10.5281/zenodo.19472999 (Fig2C-FISH_infection_rate.xlsx).

**Fig 3:**
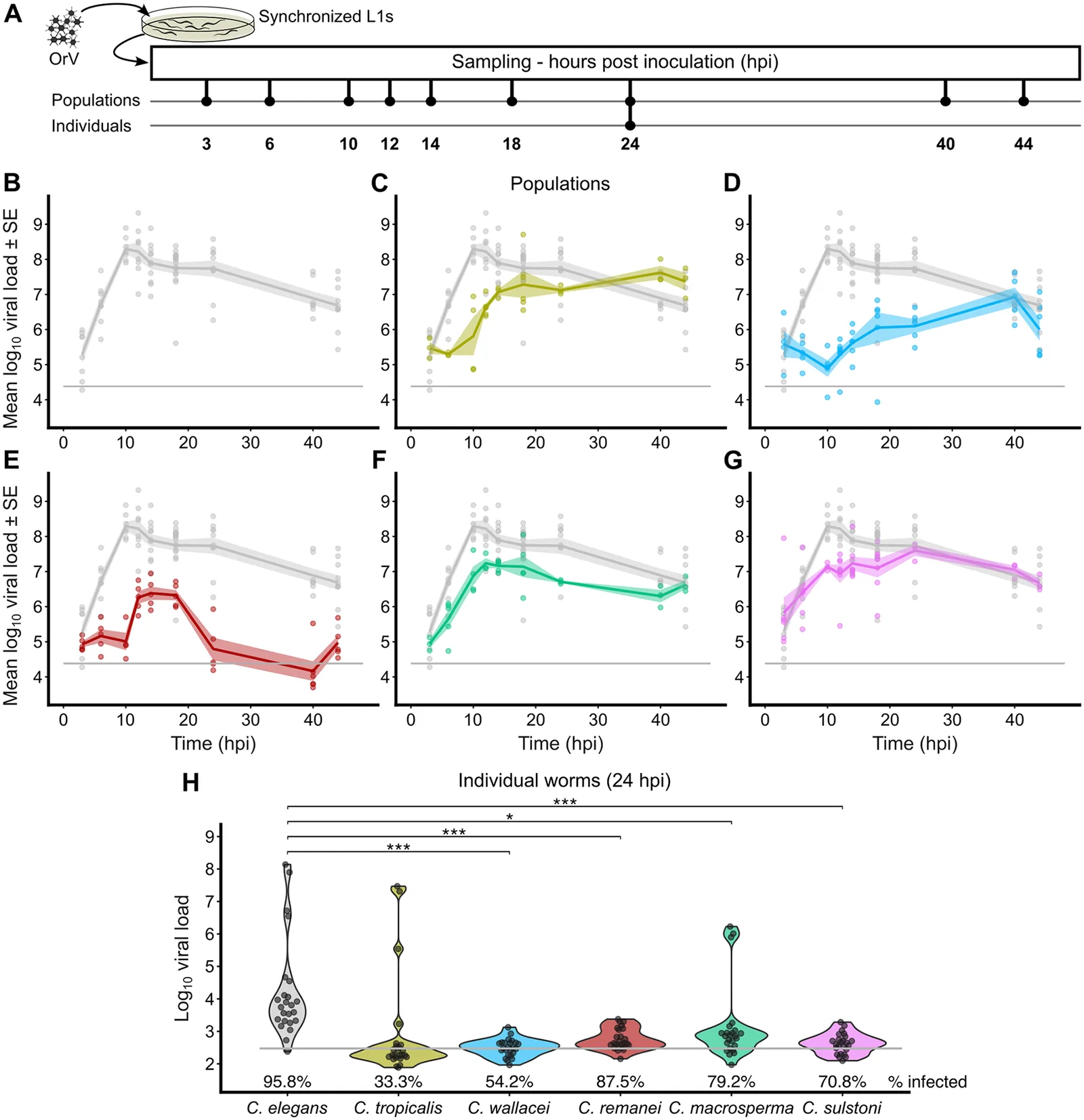
OrV viral load dynamics and single worm viral load distributions across host species. **(A)** Schematic explanation of the experimental design. Synchronized populations of ~500 worms were inoculated at hatching with 2.8 × 10^9^ copies of OrV RNA2 and sampled at the indicated timepoints for total RNA extraction or single worm lysis and OrV viral load by measured by absolute RT-qPCR. Viral load over time throughout the first 44 hpi in the different host species: **(B)** *C. elegans* ERT54 (gray), **(C)** *C. tropicalis* (yellow), **(D)** *C. wallacei* (blue), **(E)** *C. remanei* (red), **(F)** *C. macrosperma* (green), and **(G)** *C. sulstoni* (pink). Viral load was measured as the number of OrV RNA2 copies in 10 ng of total RNA; 3–16 replicates per strain per time point. **(H)** Violin plots of OrV viral load of individual animals (*n* = 24 for each species) at 24 hpi. Viral load was measured as the number of OrV RNA2 copies in 1/5 of the single worm lysate. Horizontal gray lines represent the technical detection limit (mean + 2 SD of negative controls). The data underlying this Figure can be found in https://doi.org/10.5281/zenodo.19472999 (Fig3B-G-population_viral_load_curves.xlsx, Fig3H_FigS4-individual_viral_load.xlsx).

Viral RNA was detected at 24 hpi in every host species except *C. remanei*, in which positive smiFISH signal was observed only at 14 hpi (Fig 2A and 2B), consistent with the viral accumulation dynamics shown in Fig 3E (see Barrier 2 below). Host species had a significant effect on the proportion of animals with detectable viral RNA (Fig 2C; 24 hpi: *χ*^2^ = 308.571, 4 d.f., *P* < 0.0001; 14 hpi: *χ*^2^ = 223.319, 1 d.f., *P* < 0.0001). At 24 hpi, *C. elegans* showed the highest proportion of smiFISH-positive animals (64.0%), whereas all other species exhibited significantly lower rates of detectable viral RNA: *C. tropicalis* 6.3%, *C. wallacei* 1.8%, *C. macrosperma* 4.9%, and *C. sulstoni* 9.3% (*P* < 0.0001 for all comparisons). At 14 hpi, viral RNA was detected in only 5.2% of *C. remanei* animals, again significantly lower than in *C. elegans* at the same time point (79.5%, *P* < 0.0001).

These differences indicate that alternative host species vary strongly in the frequency with which OrV RNA is detected after exposure. However, reduced smiFISH positivity could reflect one or more early bottlenecks, including inefficient entry, impaired uncoating, failure to initiate or sustain replication, or rapid elimination of viral RNA by the host. Although smiFISH detects positive-strand viral RNA rather than active replication directly, previous work indicates that detectable signals require substantial viral RNA accumulation [39]. We therefore considered smiFISH positivity together with viral-load time courses, RNA1 and RNA2 co-detection, viral localization, and transmission assays to infer whether infection progressed beyond early viral RNA detection.

Consistent with impaired early-cycle progression in some hosts, we also observed substantial fractions of RNA1-positive cells or animals lacking detectable RNA2 at 14 hpi, particularly in *C. remanei* (see Barrier 3 below). This pattern is consistent with incomplete progression to balanced accumulation of both viral genome segments and may reflect abortive early infection and/or rapid host clearance. Together, these results support the existence of strong early barriers to productive OrV infection in several host species.

### Barrier 2: Within-host replication dynamics

To better understand the replication dynamics of OrV in the alternative hosts, we measured viral load during the first 44 hpi. For this purpose, synchronized populations of ~500 animals were inoculated at hatching and sampled at nine time points (Fig 3A). Viral load was quantified as the number of OrV RNA2 copies per ng of total RNA using the absolute RT-qPCR method. The viral load trajectory observed in *C. elegans* (Fig 3B) matched our previous results [39]. Among the different host species, viral load dynamics differed significantly, as did the interaction between host species and time (Fig 3B–3G; gray horizontal line indicates the technical detection cutoff; *χ*^2^ = 281.081, 4 d.f., *P* < 0.0001; *χ*^2^ = 302.444, 48 d.f., *P* < 0.0001, respectively).

Averaged across all time points, *C. elegans* exhibited the highest viral load of all host species (*C. tropicalis*, −77.4%, *P* < 0.0001; *C. wallacei*, −97.0%, *P* < 0.0001; *C. remanei*, −98.9%, *P* < 0.0001; *C. macrosperma*, −82.7%, *P* < 0.0001; and *C. sulstoni*, −59.6%, *P* = 0.0008). Moreover, the alternative hosts showed differences in the duration of the eclipse phase of viral accumulation compared to the natural host. Slopes estimated from an exponential growth model using the first 10 hpi were lower for all alternative hosts relative to *C. elegans* (*C. tropicalis*, −86.9%, *P* = 0.0001; *C. wallacei*, −123.3%, *P* < 0.0001; *C. remanei*, −97.4%, *P* < 0.0001; *C. macrosperma*, −34.5%, *P* = 0.0317; and *C. sulstoni*, −56.0%, *P* = 0.0089). These results suggest a possible desynchronization of the viral replication cycle, with a prolonged lag phase and/or slower exponential growth rate in the alternative hosts. Viral load remained high throughout the entire 44 hpi observation period in all species except *C. remanei*, which showed only a short replication period followed by viral clearance at 24 hpi. Overall, these results indicate that OrV replication occurs with species-specific dynamics that are slower, reach lower peaks, and reflect a temporal shift in the viral cycle relative to the natural host, *C. elegans*.

To further examine inter-individual differences in the ability of *C. elegans* and alternative hosts to support viral replication and accumulation, we inoculated synchronized animals at hatching and measured the viral load of individual animals at 24 hpi. After discarding individuals with values below the cutoff threshold (mean + 2 SD of RT-qPCR values from noninfected animals), we found significant differences in viral load among host species (Fig 3H; *χ*^2^ = 30.757, 4 d.f., *P* < 0.0001). *C. elegans* and *C. tropicalis* formed a homogeneous group with higher viral load than the remaining four species (*C. wallacei*, −97.7%, *P* = 0.0001; *C. remanei*, −96.7%, *P* = 0.0001; *C. macrosperma*, −88.5%, *P* = 0.0389; *C. sulstoni*, −97.1%, *P* = 0.0001).

Viral load among individuals of the same species showed significantly right-skewed distributions in all cases: *C. elegans* (range: 5.3 × 10^2^–1.4 × 10^8^; *g*_1_ = 3.537 ± 0.481, *P* < 0.0001), *C. tropicalis* (3.2 × 10^2^–2.9 × 10^7^; *g*_1_ = 1.611 ± 0.752, *P* = 0.0322), *C. wallacei* (3.0 × 10^2^–1.3 × 10^3^; *g*_1_ = 2.368 ± 0.616, *P* = 0.0001), *C. remanei* (3.0 × 10^2^–2.4 × 10^3^; *g*_1_ = 1.247 ± 0.501, *P* = 0.0129), *C. macrosperma* (3.0 × 10^2^–1.69 × 10^6^; *g*_1_ = 2.552 ± 0.524, *P* < 0.0001), and *C. sulstoni* (3.1 × 10^2^–1.9 × 10^3^; *g*_1_ = 1.857 ± 0.550, *P* = 0.0007). To test whether bimodal Lognormal models better fit our data than a unimodal single Lognormal distribution, we compared fits between the two approaches (Table 1, Fig A in S1 Appendix). Based on BIC evidence weights (>0.95), viral load data from *C. wallacei* and *C. sulstoni* were well explained by single Lognormal distributions (Fig A in S1 Appendix, panels C and F). In contrast, model fit improved significantly for *C. elegans*, *C. tropicalis* and *C. macrosperma* when using a mixture of two Lognormals (Fig A in S1 Appendix, panels A, B and E), despite the addition of three parameters (Table 1). Statistical power was not enough to decide among models in the case of *C. remanei* (Fig A in S1 Appendix, panel D). For *C. elegans*, 83.3% of individuals fell into a distribution with a mean of 4.0 × 10^3^, whereas the remaining high producers followed a distribution with a mean of 2.1 × 10^7^. For *C. tropicalis*, 87.5% belonged to a distribution with a mean of 2.1 × 10^2^, and high producers followed a distribution with a mean of 5.9 × 10^6^. For *C. macrosperma*, 87.5% produced low viral loads (mean 5.2 × 10^2^), while high producers followed a distribution with a mean of 1.1 × 10^6^. These results suggest that the abundance of high producers varies across species, having a direct consequence on the differences in the prevalence of infections among host species.

**Table 1:** Fitting of viral loads (VL) to unimodal or bimodal Lognormal models.

Species	Lognormal model	BIC	BIC evidence weights	*p*_1_	Mean_1_(logVL)	SD_1_(logVL)	Mean_2_(logVL)	SD_2_(logVL)
*C. elegans*	Unimodal	600.461	0.0005	1	9.717	3.460		
	Bimodal	585.120	0.9995	0.833	8.288	1.216	16.859	1.619
*C. tropicalis*	Unimodal	453.026	0.0000	1	6.629	3.518		
	Bimodal	406.749	1.0000	0.875	5.348	0.655	15.591	2.028
*C. wallacei*	Unimodal	325.608	0.9885	1	5.735	0.605		
	Bimodal	334.507	0.0115	0.114	4.845	0.220	5.849	0.540
*C. remanei*	Unimodal	364.578	0.5910	1	6.356	0.732		
	Bimodal	365.314	0.4090	0.711	5.946	0.375	7.366	0.273
*C. macrosperma*	Unimodal	467.357	0.0000	1	7.221	2.622		
	Bimodal	427.781	1.0000	0.875	6.264	0.717	13.922	0.314
*C. sulstoni*	Unimodal	346.774	0.9775	1	6.035	0.697		
	Bimodal	354.316	0.0225	0.933	5.932	0.603	7.449	0.125

*p*_1_ is the weight of the first Lognormal distribution describing the low viral load individuals. 1 − *p*_1_ thus corresponds to the weight of the second Lognormal describing the high viral load individuals.

Abbreviation: BIC, Bayes information criterion.

### Barrier 3: Viral genome stoichiometry and life-cycle progression

**Fig 4:**
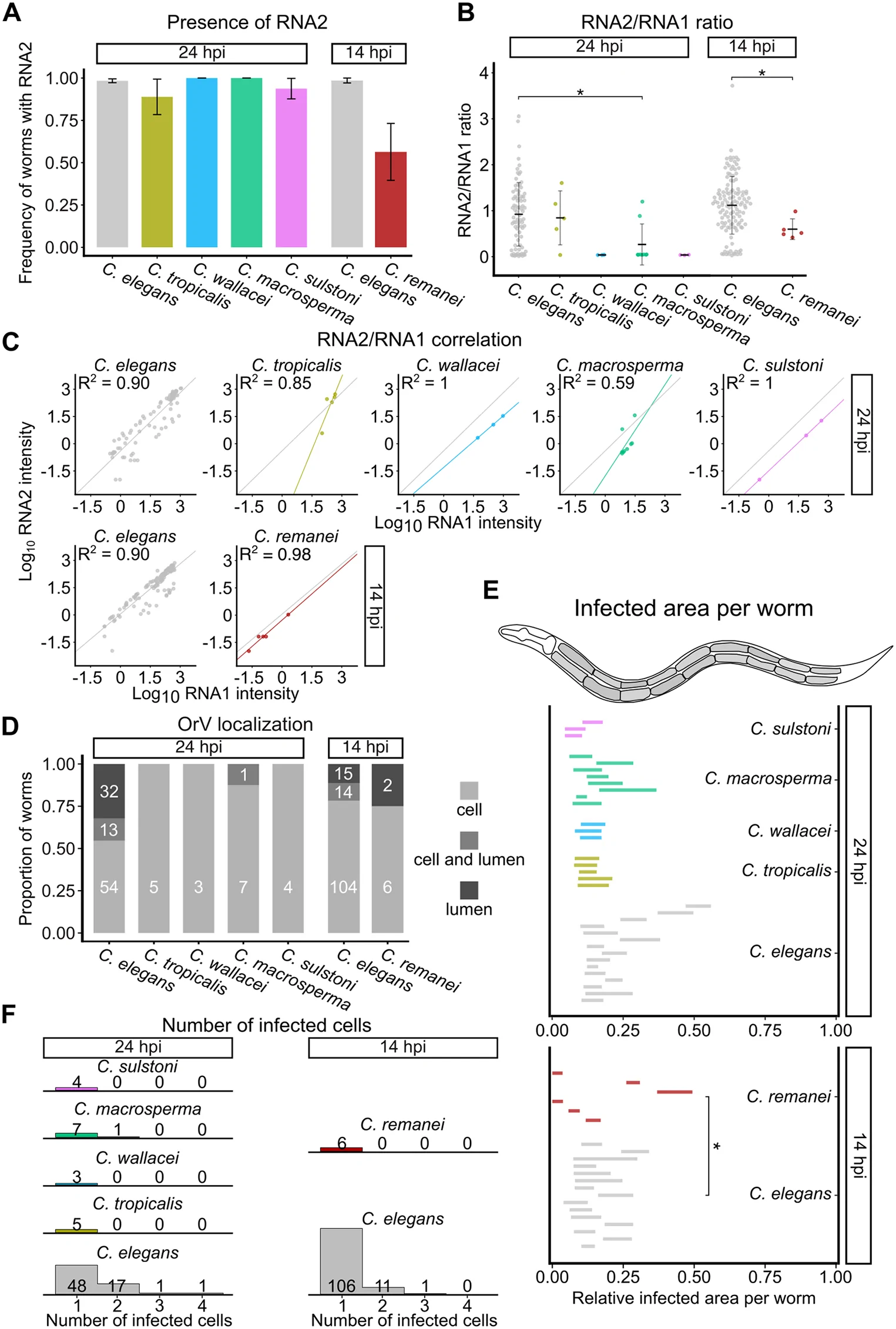
Spatial localization and RNA ratios of OrV infection in alternative host species. **(A)** Bar plot of the proportion of animals in which OrV RNA2 signal was detected. Error bars represent the standard error of the Binomial proportion (*C. elegans*, gray; *C. tropicalis*, yellow; *C. wallacei*, blue; *C. remanei*, red; *C. macrosperma*, green; *C. sulstoni*, pink). **(B)** OrV RNA2:RNA1 ratios at 24 and 14 hpi. OrV RNA2:RNA1 ratios were calculated based on the normalized median intensity values of RNA1 and RNA2 smiFISH signal. **(C)** Regression plots of RNA1 and RNA2 intensities in each species and time point. As a null model, the regression line of the corresponding *C. elegans* time point is shown as a gray line in all panels. Deviations from the null model were evaluated using BIC evidence weights (Table A in S1 Appendix). Intensities are displayed in arbitrary units. **(D)** OrV localization within cells, intestinal lumen, or cell and lumen at 24 and 14 hpi. Bar plot shows the proportion of infected worms in each category within each species and time point. The numbers inside the bar plot denote the number of worms in each category. **(E)** Infected area and position of infection per animal relative to intestine length at 24 and 14 hpi. At 14 hpi, *C. elegans* and *C. remanei* differ significantly in the size of the relative infected area (10.4% vs. 5.7%, *P* = 0.0014). **(F)** Number of infected cells per animal at 24 and 14 hpi. The histograms represent the relative abundance of each category within each species and time point. The data underlying this Figure can be found in https://doi.org/10.5281/zenodo.19472999 (Fig4A-presence_of_RNA2.xlsx, Fig4B-C-RNA2_RNA1_ratios.xlsx, Fig4D_F-cell_lumen_localization_and_number_of_cells.xlsx, Fig4E_relative_area_and_position.xlsx).

To clarify the stage of the viral cycle reached in each host species, we further analyzed our previously obtained smiFISH dataset by determining whether infected animals contained only RNA1 or both viral RNA segments. Overall, most infected animals contained both RNAs, and all animals with viral signal in the intestinal lumen carried both RNA segments. However, at 14 hpi, RNA2 was undetectable in a substantially higher fraction of infected *C. remanei* animals than of infected *C. elegans* animals. Specifically, 4/9 infected *C. remanei* animals contained only RNA1, compared with 4/138 infected *C. elegans* animals (Fig 4A; Fisher’s exact test, *P* = 0.0004; odds ratio = 0.050). This enrichment of RNA1-positive/RNA2-negative infections in *C. remanei* suggests that OrV infection in this species frequently fails to progress to balanced accumulation of both viral genome segments, consistent with early-cycle failure or rapid clearance. By 24 hpi, RNA2-negative infections were uncommon among the species in which infected animals were detected (*C. elegans*, 2/123; *C. tropicalis*, 1/9; *C. wallacei*, 0/3; *C. macrosperma*, 0/8; and *C. sulstoni*, 1/16), and their frequency did not differ significantly among species (Fisher’s exact test, *P* = 0.2634). Thus, the clearest species-specific pattern was the elevated frequency of RNA1-only infections in *C. remanei* at 14 hpi, supporting the idea that OrV infection is often abortive or transient in this host.

We previously showed that the RNA2:RNA1 ratio changes dynamically during infection in *C. elegans* [39]. As an additional measure of viral cycle progression, we quantified the RNA2:RNA1 ratio in each species by comparing their respective normalized smiFISH fluorescence intensities. RNA2:RNA1 ratio was significantly higher in *C. elegans* (1.1 ± 0.6) than in *C. remanei* (0.6 ± 0.2) at 14 hpi (Fig 4B; 46.3%, *P* = 0.0228). At 24 hpi, overall significant differences in the ratio were also found (Fig 4B; *χ*² = 16.423, 4 d.f., *P* = 0.0025) among species, with *C. elegans* still showing the highest mean ratio (0.92 ± 0.07). However, pairwise differences between *C. elegans* and *C. macrosperma* (0.3 ± 0.2) showed the only significant case (*P* = 0.0345). (In all other comparisons: *C. tropicalis* 0.8 ± 0.3, *P* = 1; *C. wallacei* 0.04 ± 0.37, *P* = 0.1652; and *C. sulstoni* 0.04 ± 0.37, *P* = 0.0512.) The measured RNA1 and RNA2 intensities were strongly correlated after controlling for host species and time post-inoculation (Fig 4C; *r_p_* = 0.787, 232 d.f., *P* < 0.0001). Most of the cells of the alternate species fell below the *C. elegans* regression line (shown in gray), indicating that the ratio of RNA2:RNA1 in these hosts is skewed towards a greater amount of RNA1 compared to the *C. elegans* average (in all cases, BIC evidence weights ≥ 0.8495 for the fitting to a regression line different from the one observed for *C. elegans*; Table A in S1 Appendix). Only a few cells in *C. macrosperma* and *C. tropicalis*, the two species with high-viral load individuals, fell above the *C. elegans* line; however, all observed values were within the variability of observations in *C. elegans*. These results indicate that, depending on the host species, OrV is less efficient at replicating RNA2 relative to RNA1 and therefore may be impaired in producing new virions.

### Barrier 4: Virion egress and tissue localization

To further assess how far OrV infection progressed in each host species, we examined the localization of viral RNA signal in the smiFISH dataset. Infected animals were classified according to whether viral RNAs were detected within intestinal cells, in the intestinal lumen or in both compartments (Fig B in S1 Appendix, panel A). Detection of viral RNA in the lumen was used as a proxy for progression beyond intracellular RNA accumulation toward a potentially transmissible stage. At 24 hpi, viral RNA localization differed significantly among species (Fig 4D; *χ*^2^ = 10.073, 3 d.f., *P* = 0.0180). In *C. elegans*, many infected animals showed luminal viral RNA, either exclusively in the lumen (32/99) or in both cells and lumen (13/99). By contrast, infection in the other species was predominantly cell-restricted, with luminal viral RNA detected only in one *C. macrosperma* individual (cell + lumen: 1/8). In *C. elegans*, luminal localization was less frequent at 14 hpi than at 24 hpi (14 hpi: lumen, 15/133; cell + lumen, 14/133; Fig 4D; *χ*^2^ = 16.552, 1 d.f., *P* < 0.0001), consistent with luminal viral RNA reflecting a later stage of infection. Although two *C. remanei* individuals showed luminal viral RNA at 14 hpi, this pattern was not observed at 24 hpi. Thus, rather than indicating desynchronization per se, these data suggest that OrV infection in most alternative hosts rarely progresses to detectable luminal viral RNA, consistent with impaired completion of later steps such as packaging, egress, or maintenance of transmissible infection.

To complement localization, we quantified infected area and position along the intestine and number of infected cells per animal. At 24 hpi, no significant differences were detected among species in relative infected area (Fig 4E; ~10.1% of the measured area infected, *χ*^2^ = 3.079, 3 d.f., *P* = 0.3796), and no significant positional differences were observed either (*χ*^2^ = 7.502, 3 d.f., *P* = 0.0575). At 14 hpi, the infected area in *C. remanei* (5.7%) was smaller than in *C. elegans* (10.4%) (*P* = 0.0143), although no significant positional differences were observed (*P* = 0.6222). The number of infected cells did not differ significantly among species at 24 hpi (Fig 4F; *χ*^2^ = 0.680, 3 d.f., *P* = 0.8780) or between *C. elegans* and *C. remanei* at 14 hpi (Fig 4F; *χ*^2^ = 0.065, 1 d.f., *P* = 0.7992). However, individuals containing two infected cells were most common, and animals with three or four infected cells were observed only in *C. elegans*, not in any alternative hosts (Fig B in S1 Appendix), mirroring the higher viral load outliers observed more frequently in *C. elegans* (Barrier 2).

### Barrier 5: Transmission competence

**Fig 5:**
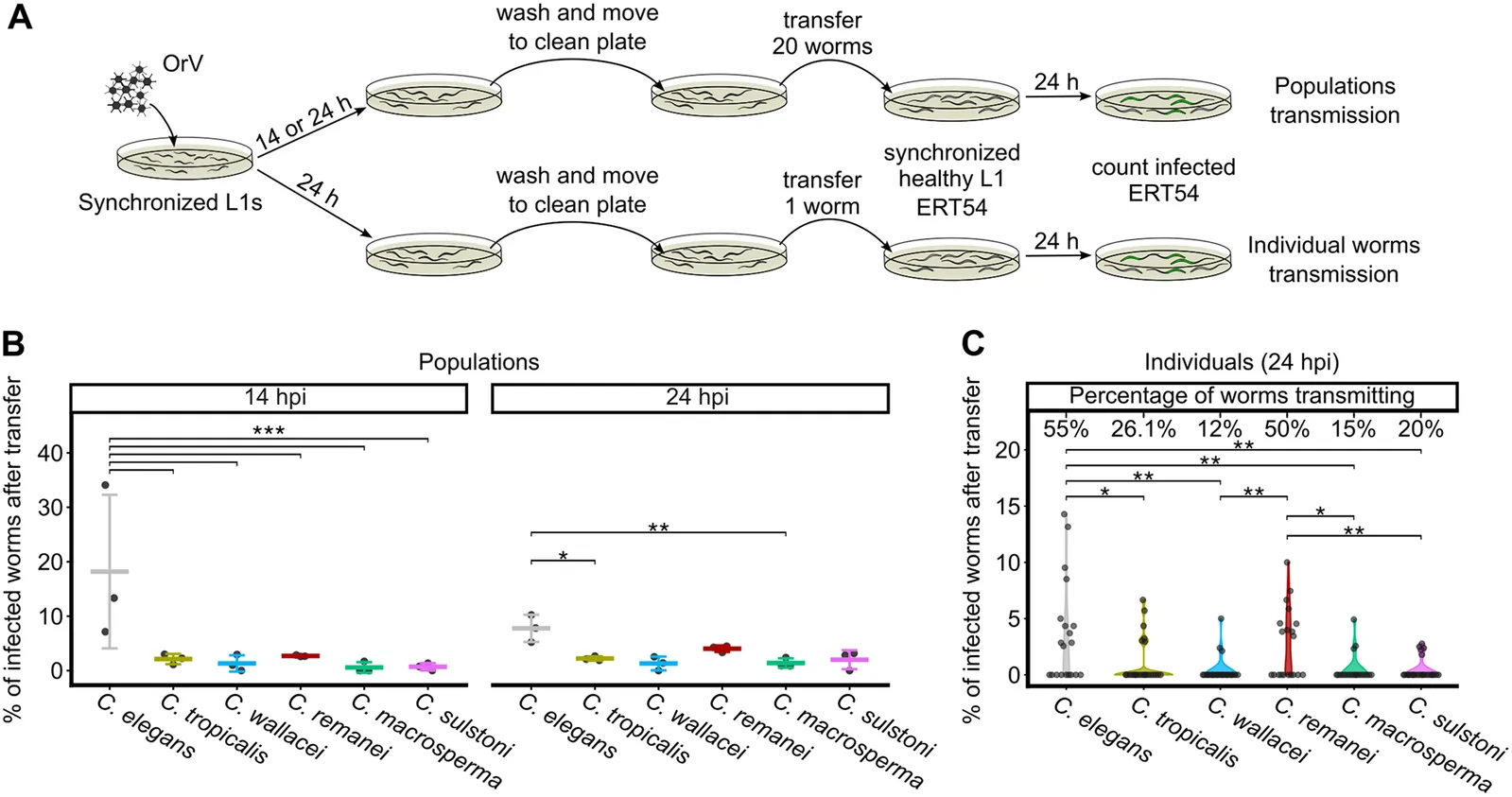
OrV transmission rates are time- and species-specific. **(A)** Experimental design. Synchronized populations of inoculated animals were washed and populations of 20 worms or individual worms were transferred to healthy *C. elegans* ERT54 animals which contain a GPF fluorescent marker of infection. Twenty-four hours after the transfer, the percentage of infected ERT54 animals was determined by counting the number of animals expressing the infection reporter. **(B)** Transmission of OrV from inoculated populations of 20 worms (*n* = 3) of different host species (*C. elegans*, gray; *C. tropicalis*, yellow; *C. wallacei*, blue; *C. remanei*, red; *C. macrosperma*, green; *C. sulstoni*, pink) to populations of 111 ± 26 healthy *C. elegans* ERT54 at 14 and 24 hpi. **(C)** Transmission of OrV from individual worms of different host species (*n* = 22 ± 3) to populations of 38 ± 15 healthy *C. elegans* ERT54 at 24 hpi. The values above each violin plot denotes the percentage of individual worms that successfully transmitted OrV. The data underlying this Figure can be found in https://doi.org/10.5281/zenodo.19472999 (Fig5B-population_transmission.xlsx, Fig5C-individual_transmission.xlsx).

To investigate how differences in OrV replication and viral cycle dynamics affect epidemiological parameters in alternative host species, we measured the probability of OrV transmission from each host species to *C. elegans*. To do this, we transferred 20 inoculated, synchronized animals at 14 or 24 hpi into populations of 111 ± 26 healthy *C. elegans* ERT54 animals (Fig 5A). This design imposed a standardized donor-population size across host species, allowing direct comparison under identical exposure conditions. The two time points were selected because they correspond to the initial release of new OrV virions (14 hpi) and a more advanced stage of the viral cycle near clearance (24 hpi) in *C. elegans* [39]. The ERT54 strain contains an integrated fluorescent infection reporter that enables real-time tracking of infections by fluorescence microscopy [61]. Twenty-four hours after transfer, we quantified the percentage of ERT54 animals expressing the infection reporter.

Transmission from groups of 20 donors differed significantly among host species (Fig 5B; *χ*^2^ = 80.061, 4 d.f., *P* < 0.0001). We also detected a significant interaction between species and the age of the infected donor animals (*χ*^2^ = 24.886, 6 d.f., *P* = 0.0004), largely driven by a pronounced decrease in transmission between 14 and 24 hpi in *C. elegans* (18.7% versus 7.4%, *P* = 0.0003). At 14 hpi, *C. elegans* exhibited the highest transmission probability (18.7%; *C. tropicalis*, 2.2%, *P* < 0.0001; *C. wallacei*, 1.2%, *P* < 0.0001; *C. remanei*, 2.7%, *P* < 0.0001; *C. macrosperma*, 0.6%, *P* < 0.0001; and *C. sulstoni*, 0.8%, *P* < 0.0001). By 24 hpi, however, transmission rates across species were more uniform, with significant differences remaining only between *C. elegans* and *C. wallacei* (1.4%, *P* = 0.0122) and *C. elegans* and *C. macrosperma* (1.4%, *P* = 0.0062).

To complement the population-donor assay and directly test whether individual animals could transmit OrV, we then transferred single donors at 24 hpi into populations of 38 ± 15 healthy ERT54 animals (Fig 5A). This assay avoids uncertainty about how many infected animals were present within each 20-donor group, although it still captures transmission only under the specific time point and experimental conditions tested. The proportion of individuals successfully transmitting infection differed significantly among host species (Fig 5C; *χ*^2^ = 13.321, 4 d.f., *P* = 0.0098). These differences reflected two statistically homogeneous groups: a higher-transmission group composed of *C. elegans* (55.0%), *C. tropicalis* (26.1%), *C. remanei* (50.0%), and *C. sulstoni* (20.0%) (*P* ≥ 0.1828 for all comparisons within the group), and a lower-transmission group composed of *C. wallacei* (12.0%) and *C. macrosperma* (15.0%) (*P* ≤ 0.0205). Host species also significantly influenced transmission outcomes (mean fraction of infected recipients; *χ*^2^ = 37.272, 4 d.f., *P* < 0.0001). *C. elegans* showed significantly higher mean transmission (3.6%) compared to all alternate hosts, except for *C. remanei* (Fig 5C; *C. tropicalis*, 1.1%, *P* = 0.0286; *C. wallacei*, 0.3%, *P* = 0.0004; *C. remanei*, 2.9%, *P* = 1; *C. macrosperma*, 0.6%, *P* = 0.0031; *C. sulstoni*, 0.5%, *P* = 0.0017).

Together, these results show that OrV transmission, like viral load, is strongly species-specific and exhibits substantial within-species variability under the assay conditions used here. The 20-donor assay provides a standardized comparison across hosts but does not necessarily estimate the maximum transmission capacity of each species, particularly for low-prevalence hosts in which infected donors may be rare. The single-worm assay complements this approach by testing whether individual animals can transmit OrV. Combining these results with our viral load data indicates that RNA accumulation and transmission are not tightly correlated in all species, and that the relationship between viral accumulation and transmission varies in a species-specific way.

### Barrier 6: Virulence and host fitness effects

Although infection rates and viral loads were significantly lower in the alternative hosts, we sought to determine whether the developmental impact of OrV infection was nonetheless species-dependent. To do this, we imaged healthy and inoculated populations from hatching through 72 h and measured body length as a proxy for developmental rate. GLMs were fitted separately for each species.

**Fig 6:**
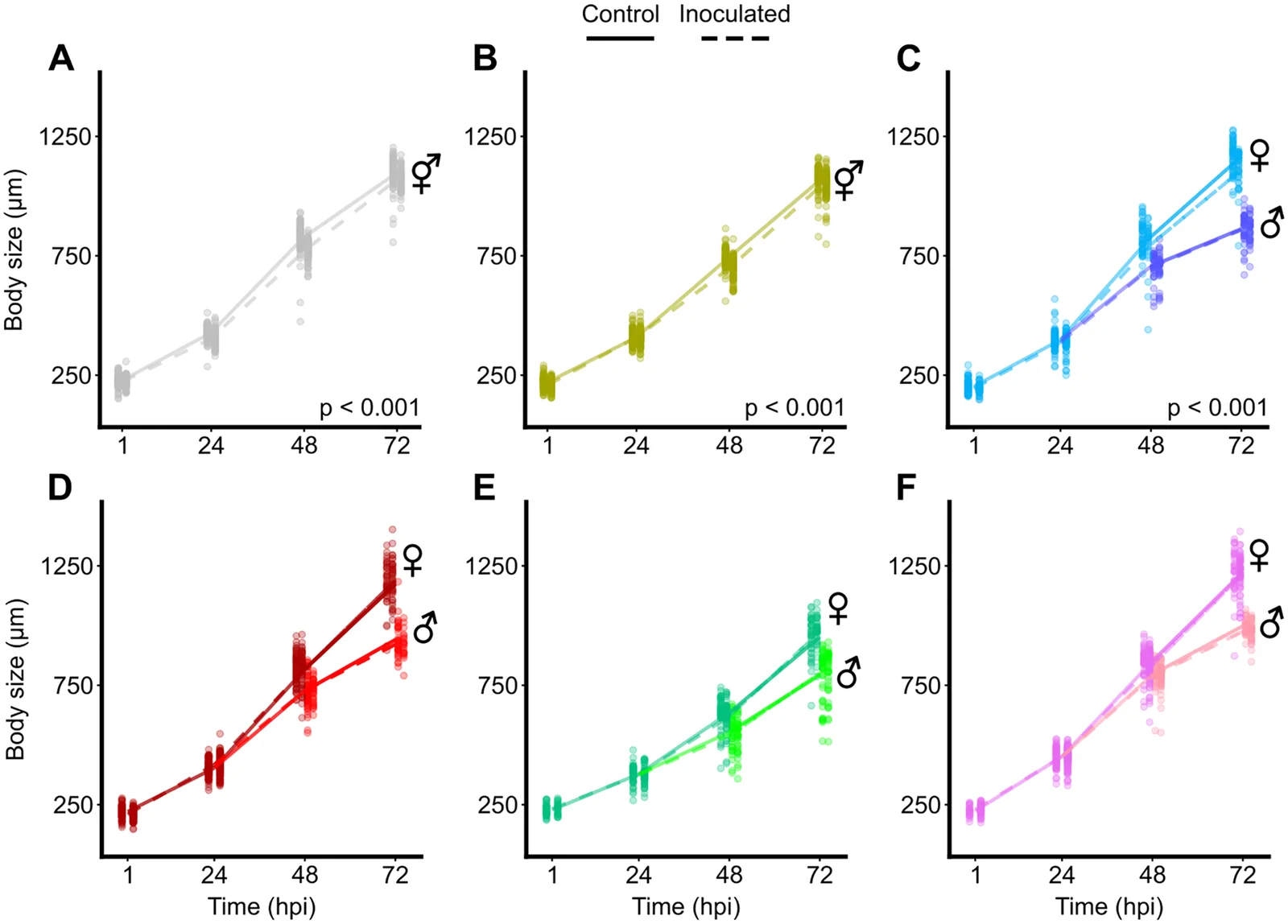
Virulence of OrV is species-specific and can change based on host sex. Virulence measured as the difference in animal body length between inoculated and control populations from hatching to 72 hours. **(A)** *C. elegans* (gray), **(B)** *C. tropicalis* (yellow), **(C)** *C. wallacei* (blue), **(D)** *C. remanei* (red), **(E)** *C. macrosperma* (green), and **(F)** *C. sulstoni* (pink). Mean body length of control populations is represented by full lines and of inoculated populations by dashed lines. Populations of gonochoristic species were separated by sex once it was clearly discernable at 48 and 72 hpi. Males of each gonochoristic species are shown in a different shade color. The sex of each population is displayed next to the corresponding line on the graph. The data underlying this Figure can be found in https://doi.org/10.5281/zenodo.19472999 (Fig6-virulence_body_length.xlsx).

For the two hermaphroditic species, *C. elegans* (Fig 6A) showed highly significant effects of infection status (*χ*^2^ = 86.535, 1 d.f., *P* < 0.0001) and the infection status-by-time interaction (*χ*^2^ = 31.261, 4 d.f., *P* < 0.0001) consistent with previous results [51]. Inoculated animals were, on average, 5.2% smaller. *C. tropicalis* (Fig 6B) showed a similar pattern, with both infection status (*χ*^2^ = 57.286, 1 d.f., *P* < 0.0001) and its interaction with time (*χ*^2^ = 38.959, 3 d.f., *P* < 0.0001) having strong effects on development. In this species, animals from inoculated plates were on average 3.4% smaller.

Because reproductive modes differ among species and sex can only be distinguished reliably from the L4 stage onward, males and females in the gonochoric species were separated at 48 and 72 h. We then tested whether sex contributed to variation in the developmental effects of OrV infection. In *C. wallacei* (Fig 6C), both infection status (infected animals 2.6% smaller; *χ*^2^ = 8.125, 1 d.f., *P* = 0.0044) and sex (males 19.6% smaller; *χ*^2^ = 416.124, 1 d.f., *P* < 0.0001) had significant effects. Infection effects remained constant across these time points (*χ*^2^ = 0.665, 1 d.f., *P* = 0.4147). Notably, a significant infection status-by-sex interaction (*χ*^2^ = 7.774, 1 d.f., *P* = 0.0053) indicated that OrV symptoms were more severe in females (4.6% reduction) than in males (0.1%).

Across the other three gonochoric species (*C. remanei*, *C. macrosperma* and *C. sulstoni*; Fig 6D–6F), sex was the primary determinant of body size. Males were consistently smaller, by 13.9% to 16.6%, and also showed slower growth, as indicated by significant sex (*χ*^2^ ≥ 160.734, 1 d.f., *P* < 0.0001) and sex-by-hpi (*χ*^2^ ≥ 21.787, 1 d.f., *P* < 0.0001) effects. In none of these species did infection status, alone or in interaction with other factors, have a detectable impact on development (*χ*^2^ ≤ 3.076, 1 d.f., *P* ≥ 0.0795 in all cases).

Together, these findings indicate that despite low infection rates in non-natural hosts, OrV infection can still exert measurable virulence at the population level.

### Barrier 7: Evolutionary sustainability

After characterizing OrV infection in the alternative host species, we tested whether OrV could be maintained through serial passage in each host under a standardized filtration-based passaging regime. Five independent viral lineages were initiated per host species by inoculating populations of 20 synchronized animals, which were allowed to grow and reproduce until plate starvation. Virus-containing material was then collected by filtration and used to inoculate the next passage. We performed this experiment using two versions of the protocol, differing in the developmental stage of the animals at inoculation, L1 or L4 (Fig C in S1 Appendix, panel A). OrV viral load in the inoculum was quantified after each passage by RT-qPCR.

When passages were initiated by inoculating L1 animals (Fig C in S1 Appendix, panel B), 4/5 OrV lineages passaged in *C. elegans* maintained detectable viral loads that fluctuated between approximately 10^4^ and 10^6^ OrV RNA2 copies/µL during passages 1–5, followed by an increase to approximately 10^7^ copies/µL at passage 6. The fifth *C. elegans* lineage showed a large decrease in viral load between passages 1 and 2, followed by persistence at low but detectable levels through passage 6. In contrast, viral loads in all lineages passaged in the alternative hosts declined sharply after passage 1 and reached levels comparable to negative controls, below approximately 10^3^ copies/µL, by passage 3 in *C. macrosperma* and by passage 4 in *C. tropicalis* and *C. wallacei*.

A similar pattern was observed when passages were initiated by inoculating L4 animals (Fig C in S1 Appendix, panel C). All OrV lineages passaged in *C. elegans* maintained high viral loads of approximately 10^6^ copies/µL across the three passages performed with this protocol. By contrast, all lineages passaged in the alternative hosts again declined to low viral-load levels, approximately 10^3^ copies/µL or below, by passage 2 and continued to decrease thereafter.

Together, these results indicate that, under our filtration-based serial passage conditions, OrV was sustainably maintained in *C. elegans* but not in the alternative host species tested. We do not interpret this outcome as demonstrating an absolute inability of OrV to adapt to, or persist in, alternative hosts. Rather, failure to maintain viral load in these species may reflect the combined effects of reduced infection prevalence, inefficient progression to transmissible stages, transmission bottlenecks, inoculum dose, the number of donor animals, timing of collection, or the selective features of the filtration-based passaging regime itself. In particular, because this protocol transfers virus recovered from filtered material, it likely favors viral genotypes that are efficiently released into the medium, and may underrepresent host-associated virus that remains within cells, within the intestinal lumen, or associated with animals at the time of collection.

We therefore conclude that the alternative host species functioned as evolutionary sinks for OrV under the specific experimental passaging conditions used here. This result is consistent with the earlier infection, viral load, localization, and transmission assays, which together suggest that infection and transmission in these hosts were insufficient to sustain OrV persistence during filtration-based passaging. However, alternative passage designs, such as transferring infected animals directly or using worm-picking regimes, could impose different bottlenecks and selective pressures by carrying host-associated virus, host traits, or different viral compartments across passages. Such approaches may therefore yield different outcomes and would be required to test whether OrV adaptation to alternative hosts is possible under less restrictive transmission regimes.

**Fig 7:**
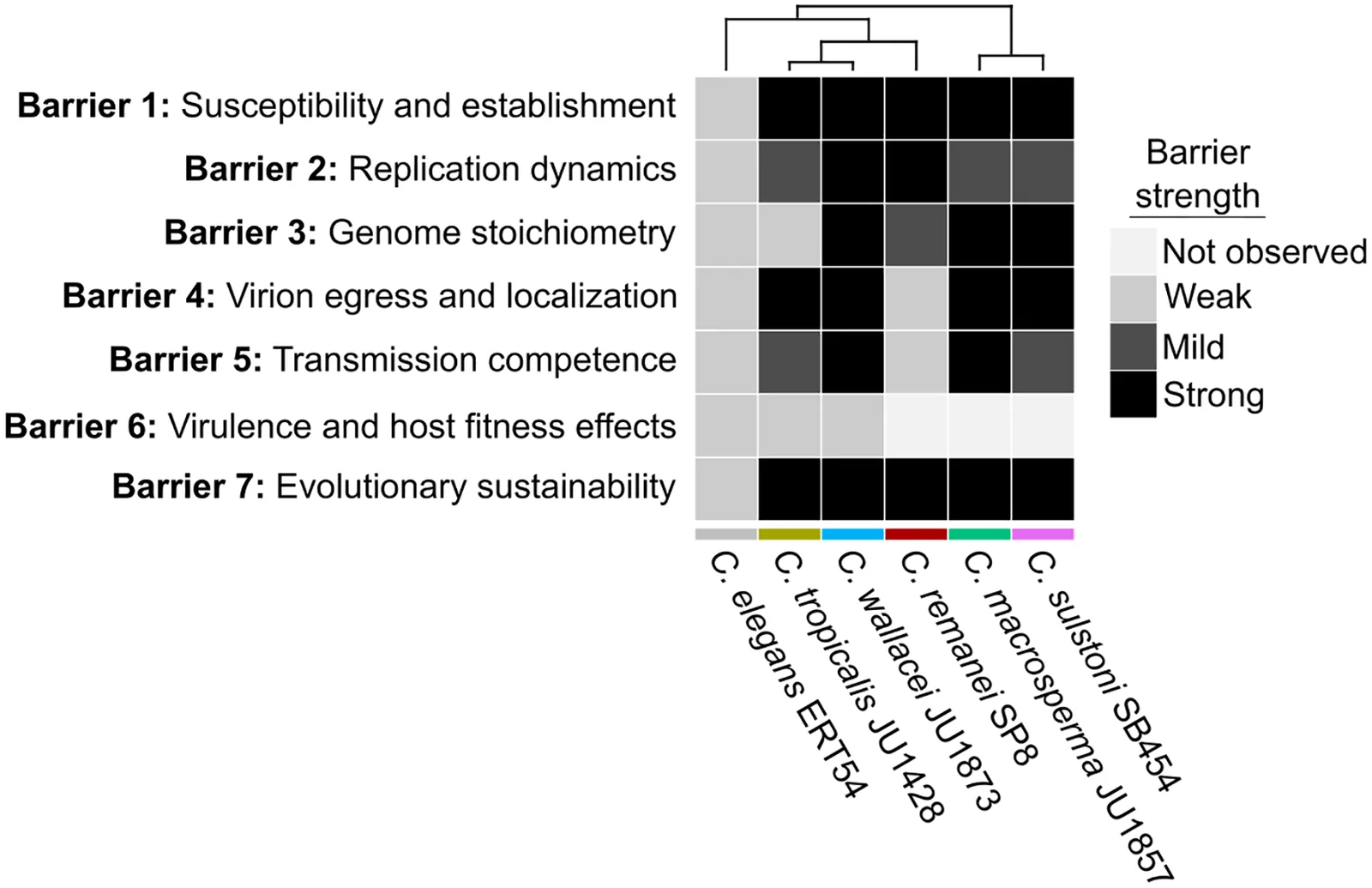
Quantitative summary of barrier strength by host species. Colors indicate whether each barrier was weak or not apparent, partial or time-limited, strongly restrictive, or not detected/not directly interpretable as a barrier metric. Categories are intended as qualitative summaries of the experimental results rather than independent statistical classes. The dashed outline around *C. remanei* highlights that mechanistic interpretation should be cautious because the number of smiFISH-positive animals was low.

Collectively, mapping infection outcomes onto the barrier framework reveals species-specific restrictions at multiple stages, including susceptibility and establishment, viral accumulation, genome-segment balance, progression toward luminal localization, transmission, and maintenance during serial passage (Fig 7). These barriers help explain why OrV persists and can be serially maintained in its natural host *C. elegans*, whereas the alternative *Caenorhabditis* hosts tested here acted as evolutionary sinks under our experimental conditions.

## Discussion

Viral emergence following host range expansion depends on how well a virus’ life cycle aligns with host biology. In the spillover framework introduced in Fig 1, cross-species infection is viewed not as a single threshold event, but as progression through a hierarchy of partially permeable barriers that span entry and early establishment, intracellular replication, genome production, egress, transmission, virulence, and evolutionary persistence [1,2,4]. The barrier summary in Fig 7 provides the empirical counterpart to this conceptual framework, showing how each *Caenorhabditis* species restricts OrV at different points along this sequence. Together, these two figures frame the main conclusion of our study: host competence is determined by the cumulative outcome of multiple life-cycle barriers, rather than by any single metric such as viral load, prevalence, or transmission alone.

By dissecting OrV infection across six *Caenorhabditis* species, we show that different hosts impose distinct combinations of restrictions that jointly determine whether infection becomes detectable, productive, transmissible, and evolutionarily sustainable, consistent with eco-evolutionary views in which emergence is constrained by bottlenecks and fitness valleys [1,56,58,62]. These restrictions occurred at multiple points along the barrier framework summarized in Fig 7. Some acted early, reducing the frequency of animals with detectable viral RNA after exposure: susceptibility varied widely across hosts, with some species showing only transient or low-frequency detection, most notably *C. remanei*, whereas others limited infection to rare individuals, such as *C. sulstoni* and *C. wallacei*. Other barriers acted downstream, after viral RNA accumulation had occurred, by altering replication dynamics, disrupting RNA2:RNA1 genome-segment balance, limiting progression toward luminal localization, or reducing onward transmission. Early-cycle failures were especially informative because RNA1-only cells in restrictive contexts suggest a possible bottleneck in which RNA2 replication or accumulation is reduced, potentially limiting virion assembly and truncating infection before it becomes efficiently transmissible. However, because the number of smiFISH-positive *C. remanei* animals was small, this interpretation should be viewed as a plausible explanation rather than a definitive mechanistic conclusion. Thus, the empirical patterns summarized in Fig 7 reveal that restrictive hosts are not equivalent dead ends: they fail at different steps, and these step-specific failures generate distinct host-competence phenotypes.

A central implication of this barrier-resolved view is that host competence is multicomponent and cannot be inferred reliably from viral load alone. Transmission assays showed that competence can be uncoupled from within-host RNA accumulation under our experimental conditions: *C. elegans* remained the most competent transmitter overall; *C. remanei* transmitted efficiently in the single-worm assay despite low accumulated RNA; and species such as *C. tropicalis* and *C. macrosperma* reached high viral loads yet showed comparatively low transmission. These discrepancies indicate that within-host amplification is necessary but not sufficient for epidemiological competence, because downstream barriers such as genome stoichiometry, egress, shedding, contact structure, and the timing of the infectious window can determine whether viral replication translates into onward spread. Importantly, the two transmission assays capture different components of this process. The 20-donor assay tests transmission in a standardized group setting, where viral production is filtered through host behavior, spatial structure, and contact patterns. This may be particularly relevant for species such as *C. wallacei* and *C. remanei*, which tend to aggregate on plates and may therefore expose recipient *C. elegans* less efficiently during the 24 h transmission window [63,64]. By contrast, the single-worm assay more directly tests whether individual animals can produce infectious virus capable of initiating infection in recipient hosts. The discrepancy between low population-level transmission and relatively high single-worm transmission in *C. remanei* therefore suggests that contact structure and behavior, in addition to viral production, can shape apparent transmission competence. These contrasting competence phenotypes mirror patterns seen in wildlife and experimental systems where susceptibility, replication, shedding, and contact behavior are often only partially correlated, and where competent hosts are defined by the joint outcome of multiple traits rather than by any single measure [55,65]. In this sense, Fig 7 extends the conceptual model in Fig 1 from a general framework into a species-resolved map of where OrV spillover is blocked, providing a mechanistic explanation for why some spillover events generate transient infections that never translate into efficient onward transmission, whereas others can propagate despite appearing weak by conventional within-host metrics [43].

Even when prevalence is low, infections can still generate measurable ecological costs, and those costs can be structured by host demography. In our system, OrV carried detectable developmental costs in *C. elegans* and *C. tropicalis*, and we also observed sex-specific effects in *C. wallacei*, emphasizing that mating system and demographic structure can modulate the population-level impact of infection [66,67]. This is consistent with the broader notion that virulence, transmission and host population structure are intertwined, such that even rare infections can alter host performance in ways relevant to ecological persistence and the opportunity for viral adaptation.

The evolutionary dimension of competence is often the most difficult to measure directly, yet it is the step that ultimately distinguishes spillover from emergence. Serial passage revealed that most alternative hosts functioned as evolutionary sinks: OrV rarely persisted beyond a few passages because prevalence, replication, and/or transmission were insufficient to sustain selection across generations. By contrast, OrV maintained higher loads and stable persistence in *C. elegans*, reinforcing the idea that a primary reservoir host is defined not merely by permissiveness, but by the ability to sustain transmission chains long enough for adaptive exploration to occur. These results place a mechanistic foundation beneath spillover theory: even if entry and replication are possible, emergence can still fail if barriers downstream, especially egress and transmission, truncate infectious periods or reduce the fraction of competent individuals to levels that prevent evolutionary persistence [1,58].

At the within-host level, OrV replication kinetics differed markedly across hosts, revealing one major route by which mismatches arise. Relative to *C. elegans*, alternative hosts tended to show prolonged eclipse phases, slower exponential growth, and lower peak loads. In many cases, viral load and transmission were correlated, but not universally so, again illustrating that replication is necessary but not sufficient for competence. In addition, single-animal measurements revealed heavy-tailed and sometimes bimodal viral load distributions, indicating pronounced within-host heterogeneity. In *C. elegans*, *C. tropicalis* and *C. macrosperma*, rare high-load individuals were associated with a higher fraction of transmitting donors, consistent with a superspreading tail in which a minority of individuals disproportionately contributes to onward transmission [54,68–70]. Yet heterogeneity alone did not determine transmission outcomes. Despite similar numbers of high-load individuals in *C. tropicalis* and *C. macrosperma*, the former transmitted more frequently than the latter, indicating that downstream barriers, particularly egress and shedding, strongly influence whether high within-host viral RNA levels translate into epidemiological competence. Conversely, we did not detect high-load outliers in *C. wallacei* and *C. sulstoni* (noting limited sample size), and transmission was correspondingly low, consistent with systems where low replication, short infectious windows, or other constraints reduce the likelihood of detectable shedding and onward transmission [71–74]. Together, these patterns underscore that competence heterogeneity can emerge both from variation in within-host amplification and from barrier-specific failures that prevent completion of the transmissible cycle even when amplification occurs.

A particularly informative barrier in this system is the stoichiometric balance between the two viral genome segments. Across species, reduced RNA2:RNA1 ratios were broadly associated with poorer progression toward transmissible infection. This pattern was clearest in *C. wallacei*, *C. macrosperma* and *C. sulstoni*, which showed strongly reduced RNA2:RNA1 ratios and also transmitted poorly in our assays. By contrast, *C. tropicalis* maintained RNA2:RNA1 ratios closer to those observed in *C. elegans* and showed comparatively better transmission than these more restrictive hosts, despite lacking detectable luminal viral RNA in the smiFISH dataset. *C. remanei* represents an important exception: although accumulated viral RNA was low and infection was transient, RNA2:RNA1 ratios in infected cells were not strongly reduced and single-worm transmission was relatively efficient. Together, these patterns suggest that balanced accumulation of RNA2 relative to RNA1 is not sufficient by itself to ensure high transmission, but that strong RNA2 under accumulation is associated with poor host competence. This provides a plausible mechanistic basis for the frequent decoupling between viral load and transmission, because high intracellular RNA accumulation may fail to generate efficient infectious output if genome-segment balance is disrupted. Genome stoichiometry therefore emerges as a barrier positioned downstream of early infection and replication, but upstream of efficient shedding and transmission, helping determine whether a host becomes epidemiologically competent.

*C. remanei* provides a potentially informative, but necessarily cautious, case. Despite low accumulated viral RNA and the absence of high-load individuals in our single-worm measurements, *C. remanei* showed measurable transmission in some assays. One possible explanation is that OrV can progress to a transmissible stage during a narrow early window in this host, as suggested by the small number of smiFISH-positive animals with lumen-localized viral RNA at 14 hpi and by relatively balanced RNA2:RNA1 ratios in infected cells. However, because the number of smiFISH-positive *C. remanei* animals was limited, we do not interpret these observations as definitive evidence for efficient egress or infectious virion production. At face value, they suggest the hypothesis that OrV infection in *C. remanei* may be brief and transient, with potential transmission occurring before viral RNA becomes undetectable at later time points. Testing this model will require higher sampling density across early time points and direct measurements of infectious virion production. More broadly, this pattern illustrates that transmission potential may depend not only on the magnitude of within-host viral RNA accumulation, but also on the timing and duration of the window during which transmissible virus is produced.

Cross-species comparisons further resolve where different hosts block the cycle. In *C. tropicalis*, RNA2:RNA1 ratios were not statistically different from *C. elegans* and loads were high, yet lumen-localized virus was not detected, suggesting that entry and/or egress (and not replication per se) constitute the principal obstacles, consistent with prior reports of high load but reduced transmission [43]. In *C. wallacei*, the absence of high-load individuals combined with uniformly low RNA2:RNA1 ratios in infected cells argues for compound barriers at entry and replication. Together with the *C. remanei* case, these outcomes show that while entry is often a hurdle across hosts [75], downstream steps such as translation, packaging, or release can be equally decisive and can differ idiosyncratically among related hosts. A natural next step is therefore to deploy recombinant OrV to bypass entry and isolate downstream constraints in each host, enabling direct tests of which barrier is rate-limiting in each competence phenotype [76].

Time-resolved measurements further highlight how barrier permeability changes across infection and how host control can act through temporal mismatch. At early time points (≤14 hpi), we detected more smiFISH-positive animals but a higher proportion of RNA1-only cells; by 24 hpi, positivity rates declined in restrictive hosts. In *C. remanei*, the combination of few smiFISH-positive animals at 14 hpi, a relatively high fraction of RNA1-only infections among those positives, and absence of detectable smiFISH signal at 24 hpi is consistent with a short-lived infection window. However, given the small number of positive animals, these data cannot distinguish whether this pattern reflects rapid clearance, incomplete viral-cycle progression, or a brief period of productive infection followed by loss of detectable viral RNA. Several traits appear virus-controlled across hosts, notably the early replication peak, whereas others are host-controlled, including the frequency of high-load outliers, the RNA2:RNA1 balance, and egress [41,77,78]. Delayed peaks in *C. tropicalis* and *C. wallacei*, together with prior evidence that replication timing depends on host developmental stage, support a major role for host physiology in shaping replication dynamics [41,50]. Conceptually, these temporal patterns reinforce the broader point that emergence can be blocked not only by absolute incompatibilities, but by mismatches in when key life-cycle steps occur relative to host development and immune clearance, mismatches that shorten infectious windows and lower the probability of sustained transmission.

Host phylogeny alone did not predict load or virulence across this genus. Although viruses often perform better in closely related hosts and virulence can correlate with host phylogeny [3,73,79], the high within-species diversity in *Caenorhabditis*, particularly in immunity genes, means that strain identity and specific host - virus interactions can dominate outcomes [47]. We observed virulence in *C. tropicalis* and *C. wallacei* despite contrasting loads, no virulence in *C. remanei* despite substantial transmission during a brief window, and no virulence in the more distant *C. macrosperma* and *C. sulstoni* despite higher loads than *C. wallacei*. These patterns indicate that virulence depends on the particular host - virus interactions that shape specific barriers (*e.g*., replication timing, tissue processes and clearance), rather than on species-level relatedness alone.

Sex-specific effects provide an additional layer of demographic structure that can shape disease impact and potentially competence. We found stronger developmental costs in females in *C. wallacei*, consistent with broader evidence that infection outcomes differ between sexes and can scale to population-level impacts [66,67,80–82]. These observations parallel reports that *C. elegans* males may be more resistant to OrV, potentially via differences in activation of antiviral programs, and they suggest that sex ratios and mating systems could modulate spillover outcomes by altering the distribution of susceptible or high-shedding individuals.

Our experimental evolution results highlight how compounded barriers and methodological choices interact to determine whether adaptation is even possible. Most alternative hosts did not sustain OrV beyond a few passages because infections were too rare, too incomplete, or too poorly transmissible, preventing the long transmission chains required for selection to act. Methodology also mattered: our filtration-based passaging selects primarily on viral traits and succeeded in *C. elegans* [40], whereas worm-picking approaches [42,43] may co-select host traits, including epigenetically regulated permissiveness [83], enabling a minority of lineages to persist under different regimes. This comparison reinforces a general eco-evolutionary point: emergence is jointly determined by viral evolvability and by the ecological and demographic conditions that permit repeated transmission, which in turn determines whether barrier-crossing mutations can be sampled and fixed.

Finally, we note that several mechanistic hypotheses involving *C. remanei* are based on a small number of smiFISH-positive animals. We therefore interpret this species as suggesting a possible narrow window of infection progression and transmission, rather than as providing definitive evidence for a specific mechanism; resolving this will require denser early time-course sampling and direct assays of infectious virion production.

In sum, the *Caenorhabditis*–OrV system reveals how entry, replication timing, genome stoichiometry, and egress interact to generate distinct host-competence phenotypes and to determine whether a host is permissive, restrictive, or an evolutionary sink. By aligning these outcomes with the spillover framework and with theory on hierarchical barriers, bottlenecks, and heterogeneity [1,43,54–56,58,62], we provide a mechanistic basis for predicting host range and emergence. More broadly, our results suggest that forecasting spillover potential will benefit from explicitly measuring barrier-specific traits, particularly the timing and completeness of egress and the stoichiometric constraints on virion production, rather than relying on viral load alone.

## Methods

### Worm strain maintenance

*C. elegans* strains ERT54 and JU2624 were obtained from E.R. Troemel and M.A. Félix, respectively. *C. elegans* N2, *C. tropicalis* JU1428, *C. macrosperma* JU1857, *C. wallacei* JU1873, and *C. sulstoni* SB454 were obtained from the Caenorhabditis Genetics Center (CGC, https://cgc.umn.edu). The genetically diverse *C. remanei* SP8 generated through artificial selection [84,85] was obtained from M. Lind. For *C. elegans*, the ERT54 strain was used in experiments unless stated otherwise. All strains were maintained under standard conditions [86,87] at 20 °C on NGM plates seeded with live *Escherichia coli* OP50. *C. elegans*, *C. tropicalis*, *C. wallacei*, and *C. remanei* belong to the *Elegans* group, while *C. macrosperma* and *C. sulstoni* belong to the *Japonica* group [53].

### OrV stock generation and quantification

OrV stock was generated by inoculating two 6 cm plates of freshly starved JU2624 worms with the OrV strain JUv1580_vlc (GenBank: PP738529.1 for RNA1 and PP738530.1 for RNA2) and expanding them on 50 9 cm NGM plates until starvation. Plates were washed with M9 buffer (0.22 M KH_2_PO_4_, 0.42 M Na_2_HPO_4_, 0.85 M NaCl, 1 mM MgSO_4_) and pooled into 15 mL tubes. Tubes were vortexed for 30 s, incubated at room temperature for 10 min, and centrifuged at 2,250*g* for 2 min. The worm pellet was discarded, and the supernatant was transferred to a new tube, centrifuged twice at 21,000*g* for 5 min at 4 °C, and filtered through a 0.22 µm syringe filter. The resulting viral preparation was stored at −80 °C.

Viral RNA was extracted using the Viral RNA Isolation Kit (NZYtech, Lisbon, Portugal) following the manufacturer’s instructions. OrV titers were quantified by standard curve RT-qPCR using the qPCRBIO SyGreen 1-Step Go Hi-ROX kit (PCR Biosystems, London, UK) on an ABI StepOnePlus Real-Time PCR System (Applied Biosystems, Foster City CA, USA), with primers targeting a region of OrV RNA2 (5′ACGAAGCAGTAGCCGTTAAG3′ and 5′GAGAACATCCTTCTCTGCGG3′).

The standard curve consisted of six serial dilutions ranging 1.25 × 10^9^–1.25 × 10^4^ copies of OrV RNA2/µL, generated from an in vitro transcribed RNA2 fragment. cDNA for in vitro transcription was synthesized using AccuScript High Fidelity Reverse Transcriptase (Agilent, Santa Clara CA, USA) and a reverse primer binding the 3′ end of RNA2 (5′ATAGCCGGGTATGGATAGCG3′). Approximately 1,000 bp from the 3′ region were amplified using a forward primer containing a 20 bp T7 promoter sequence (5′TAATACGACTCACTATAGGCCTGTCAGAGTTGAGAACA3′) and DreamTaq DNA Polymerase (Thermo Fisher Scientific, Waltham MA, USA). The PCR product was gel-purified using the MSB Spin PCRapace kit (Invitek Molecular GmbH, Berlin, Germany) and transcribed in vitro using T7 polymerase (Merck & Co., Rahway NJ, USA).

### Worm synchronization and inoculation

Synchronized worm populations were obtained by washing plates with M9 buffer to remove adults and larvae while leaving eggs behind. Plates were incubated at 20 °C for 1 h, and newly hatched larvae were collected with M9 and transferred to new plates. Larvae were inoculated by applying OrV stock containing a total of 2.8 × 10^9^ copies of RNA2 onto the *E. coli* OP50 lawns. Control plates received an equivalent volume of M9 buffer.

Unless stated otherwise, each biological replicate corresponded to an independently inoculated worm population maintained on a separate NGM plate. For assays in which multiple animals were scored from the same plate, individual animals were treated as subsamples used to estimate the plate-level outcome, such as the proportion of smiFISH-positive animals, the distribution of localization classes, or the fraction of infected recipients in transmission assays. Thus, the experimental replicate was the independently inoculated plate/population, not the individual animal alone.

### Total RNA extraction and RT-qPCR

Populations of ~500 inoculated or control worms were collected using PBS with 0.05% Tween-20. Samples were centrifuged at 400*g* for 2 min, washed three times, flash-frozen in liquid N_2_, and stored at −80 °C overnight.

For lysis, 500 µL of TRIzol (Thermo Fisher Scientific) or EasyBlue (iNtRON Biotechnology, Kirkland WA, USA) were added, followed by five freeze-thaw cycles between liquid nitrogen and 37 °C. Samples were vortexed five times for 30 s with 30 s rests. Chloroform (100 µL) was added, samples were mixed for 15 s, incubated 3 min at room temperature, and centrifuged at 16,000*g* for 15 min at 4 °C. The aqueous phase was transferred to new tubes, mixed with an equal volume of ethanol, and RNA was purified using the RNA Clean & Concentrator-5 Kit (Zymo Research, Orange CA, USA).

RNA concentration was measured with a NanoDrop OneC spectrophotometer (Thermo Fisher Scientific), and samples were diluted to 10 ng/µL. RT-qPCR was performed using 10 ng of total RNA as described above.

### Single-worm lysis and RT-qPCR

Single-worm lysis was adapted from [88]. Synchronized infected and control worms were washed three times with PBS + 0.05% Tween-20 and transferred to NGM plates without *E. coli* OP50. Individual worms were picked into 0.2 mL tubes containing 5 µL of lysis buffer (5 mM Tris pH 8.0, 0.25 mM EDTA, 0.5% Triton X-100, 0.5% Tween-20, 0.1% proteinase K). Lysis was performed in a thermocycler at 65 °C for 10 min and 85 °C for 1 min. Lysates were stored immediately at −80 °C. RT-qPCR was performed using 1 µL of lysate as template.

### Transmission assays

Synchronized, inoculated worms were washed three times with PBS + 0.05% Tween-20 and once with M9, then transferred to fresh plates. Worms were picked onto plates containing healthy *C. elegans* ERT54 worms [61]. For *C. elegans*, the N2 strain was used as donor to avoid confusion with the recipient ERT54 animals because the two strains do not show differences in viral load (Fig D in S1 Appendix; Kruskal–Wallis test, *χ*^2^ = 0.365, 1 d.f., *P* = 0.5457). After 24 h, ERT54 worms were examined for infection by GFP fluorescence using a MZ10F stereomicroscope (10 × /23B objective; mCherry M10F/MZ FLII and GFP3 MZ10F filters) (Leica Microsystems GmbH, Wetzlar, Germany). The percentage of GFP-positive ERT54 worms was calculated.

### smiFISH

The smiFISH protocol was adapted from [89,90]. Populations of ~700 inoculated and control worms were washed using the same procedure as for RNA extraction, except with one additional wash and centrifugation at 400*g* for 1 min. After the final wash, samples were fixed with 400 µL Bouin’s fixative, 400 µL 100% methanol, and 1 µL β-mercaptoethanol, mixed for 30 min at room temperature on a rotary shaker, flash-frozen, and stored at −80 °C overnight or longer.

Samples were then thawed at 4 °C for 30 min and washed four times with 1 mL borate-Triton buffer (20 mM H_3_BO_3_, 10 mM NaOH, 0.5% Triton X-100) and five times with the same buffer containing 2% β-mercaptoethanol. The last three washes included 1 h incubations at room temperature.

Samples were incubated for 5 min in wash buffer A with formamide (20% Stellaris Wash Buffer A, 20% formamide, 60% water), centrifuged, and resuspended.

Twenty-two probes targeting OrV RNA1 and 18 targeting RNA2 were designed using Oligostan [89]. Probe sequences can be found elsewhere [39–41,50]. Probes (0.83 µM) were annealed with a FLAP-label (CAL Fluor 610 or Quasar 670) at 85 °C for 3 min, 65 °C for 3 min, and 25 °C for 5 min. Hybridization was performed overnight at 37 °C in the dark.

The next day, samples were washed with wash buffer A, incubated 30 min at 37 °C, stained with 25 ng DAPI, washed with Stellaris Wash Buffer B, resuspended in 70 µL of Buffer B with 0.1 ng DAPI, and mounted with N-propyl gallate. For each replicate, 56 ± 4 animals were randomly selected and imaged using a DMi8 microscope with a DFC9000 GTC sCMOS camera (Leica Microsystems GmbH). Images were processed in Fiji [91].

Animals were counted as infected if at least one of the OrV RNAs was detected. Infected animals were analyzed by measuring the distance from the pharyngeal-intestinal junction to the start and end of the infected region. Relative infected area was calculated as the infected length divided by the total intestinal length. The median fluorescence intensity of infected cells was measured for both RNA1 and RNA2. Background values were obtained from control worms. Median fluorescence intensities were background-corrected and normalized by the number of probes used for each RNA, and RNA2:RNA1 ratios were calculated.

### Growth images and measurements

Populations of 80 ± 25 synchronized infected and control worms were transferred to NGM plates with *E. coli* OP50. Plates were imaged at designated time points using a MZ10F stereomicroscope and Flexacam C3 camera (Leica Microsystems GmbH). Worm length was measured in Fiji by tracing the midline from the anterior tip of the head to the posterior end of the body, excluding the tail.

### Experimental evolution

The first passage was started with populations of 20 synchronized L1 or L4 worms which were inoculated with 2.8 × 10^9^ OrV RNA2 copies in NGM plates seeded with 200 µL of *E. coli* OP50. The populations were allowed to grow and reproduce until plate starvation at which point worms and virus from each plate were collected with M9 buffer. Each sample was vortexed 3 times for 30 s with 30 s rests and left to incubate at room temperature for 10 min. Samples were then centrifuged for 2 min at 400*g* and 4 °C. The supernatant was then centrifuged for 5 min at 12,000*g* and 4 °C, filtered through a 0.22 µm syringe filter, and stored at −80 °C. For each subsequent passage, worms and plates were prepared as described for the first passage, however, inoculation was done with 100 µL of OrV filtrate from the previous passage. The OrV filtrate from each passage was quantified by standard curve RT-qPCR as described for OrV stock quantification above.

### Statistical analysis

Statistical analyses were performed in R version 4.5.2 using RStudio Desktop version 2026.01.0+ (Posit, Boston, MA, USA) or in SPSS version 30.0 (IBM, Armonk, NY, USA). Figure panels were organized in Inkscape version 1.2.

For population-level assays, independently inoculated plates were treated as biological replicates. When multiple animals were scored within a replicate, the data were analyzed as replicate-level counts or proportions, with the number of scored animals defining the binomial denominator where applicable. This structure avoids treating animals sampled from the same plate as independent experimental replicates.

Log_10_-transformed viral load data from population experiments were analyzed using generalized linear models (GLMs) with a Normal distribution and identity-link function. Nematode species was included as a fixed factor nested within its corresponding taxonomic group (*Elegans* or *Japonica*). Time post-infection (hpi) was incorporated as an orthogonal random factor, and the species-by-time interaction was also evaluated. For single-animal viral load estimates, hpi was not included as a factor. To characterize the heavy-tailed viral load distributions, we fitted two alternative models: (*i*) a unimodal Lognormal distribution with two parameters, and (*ii*) a mixture of two Lognormal distributions with weights *p*_1_ and 1 − *p*_1_ and four additional parameters. Model fitting was performed using the Levenberg-Marquardt algorithm.

Transmission data from population experiments were analyzed as replicate-level binomial responses using GLMs with a Binomial distribution and logit-link function, with the number of infected recipient animals and the total number of recipient animals defining the response for each independently inoculated donor population. Species was treated as a fixed factor nested within taxonomic group, and host age at inoculation (14 or 24 h) was included as an orthogonal random factor. The interaction between species and age was also tested. For single-animal transmission assays, age was not included as a factor.

Infectivity data from smiFISH experiments at 14 or 24 hpi were analyzed as replicate-level binomial responses using GLMs with a Binomial distribution and logit-link function, with the number of smiFISH-positive animals and the total number of scored animals defining the response for each independently inoculated plate. Species was treated as a fixed factor nested within taxonomic group.

The effects of nematode species on the area and relative position of infected cells along the body axis at 24 hpi were analyzed using GLMs with a Normal distribution and identity-link function, with species nested within taxonomic group as a fixed factor. Comparisons between *C. elegans* and *C. remanei* at 14 hpi were conducted using Mann–Whitney *U* tests.

Counts of infected cells per animal at 14 and 24 hpi were analyzed using GLMs with a Poisson distribution and log-link function, with species nested within taxonomic group as a fixed factor.

The presence of virus in distinct anatomical locations (cells, lumen or both) was analyzed using GLMs with a Multinomial distribution and an accumulated logit-link function, with species treated as a fixed factor nested within taxonomic group.

Ratios of normalized RNA2 to RNA1 fluorescence at 24 hpi were analyzed using GLMs with a Normal distribution and identity-link function, with species nested within taxonomic group. Comparisons between *C. elegans* and *C. remanei* at 14 hpi were performed using Mann–Whitney *U* tests.

Finally, developmental size data were analyzed by fitting GLMs with a Normal distribution and identity-link function, including infection status and hpi as orthogonal factors. Biological replicate was nested within the interaction between the two orthogonal factors. For gonochoristic species (*C. wallacei*, *C. remanei*, *C. macrosperma*, and *C. sulstoni*), gender was included as an additional orthogonal factor.

For model selection analyses, we used the Bayesian information criterion (BIC) to compare competing models while accounting for differences in model complexity. Relative support for each candidate model was quantified using BIC evidence weights, which were calculated from ΔBIC values and interpreted as the probability that a given model was the best approximating model among those considered. BIC evidence weights were used to evaluate support for unimodal *versus* bimodal Lognormal fits of single-animal viral load distributions and to compare species-specific versus *C. elegans* reference regressions in the RNA2 to RNA1 intensity analyses.

In all analyses, *post hoc* pairwise comparisons were conducted using the Holm-Bonferroni method. Additional statistical tests are described where relevant in the text.

## Supporting information

S1 Appendix Supplementary figures and table. (PDF)

## Abbreviations

CGC: Caenorhabditis Genetics Center
DIC: differential interference contrast
GLMs: generalized linear models
hpi: hours post-inoculation
OrV: Orsay virus
smiFISH: single-molecule fluorescence in situ hybridization
