# Supplementary material for: Species-specific barriers restrict virus spillover potential across the Caenorhabditis genus
Source: PLoS Biol. 2026 Aug 18;24(8):e3003965. doi: 10.1371/journal.pbio.3003965 (PMC13497278; doi:10.1371/journal.pbio.3003965)
Supplement: Supplementary file 1 [file pbio.3003965.s001.pdf]

Figure A. Distribution of viral loads of individual animals and corresponding fitted probability density functions. OrV viral load of individual animals (n = 24 for each species). (**A**) *C. elegans* (grey), (**B**) *C. tropicalis* (yellow), (**C**) *C. wallacei* (blue), (**D**) *C. remanei* (red), (**E**) *C. macrosperma* (green), and (**F**) *C. sulstoni* (pink) at 24 hpi. Viral load was measured as the number of OrV RNA2 copies in 1/5 of the single worm lysate. Horizontal and vertical grey shaded bands correspond to one standard deviation around the control-group mean viral load. First row: individual viral load measurements are shown as dots, together with a violin plot that highlights the regions of highest data density within each strain. Second row: fitted probability density function with the original viral load variable, using either a Lognormal or a bimodal Lognormal distribution depending on the strain (Table 1). Third row: the same fitted distributions shown in logarithmic space, where they correspond to normal or bimodal normal distributions for $\log_{10}$-viral load. In both cases, the displayed curves are derived from the same fitted parameters.

The data underlying this Figure can be found in https://doi.org/10.5281/zenodo.19472999 (Fig3H_FigS4-individual_viral_load.xlsx).

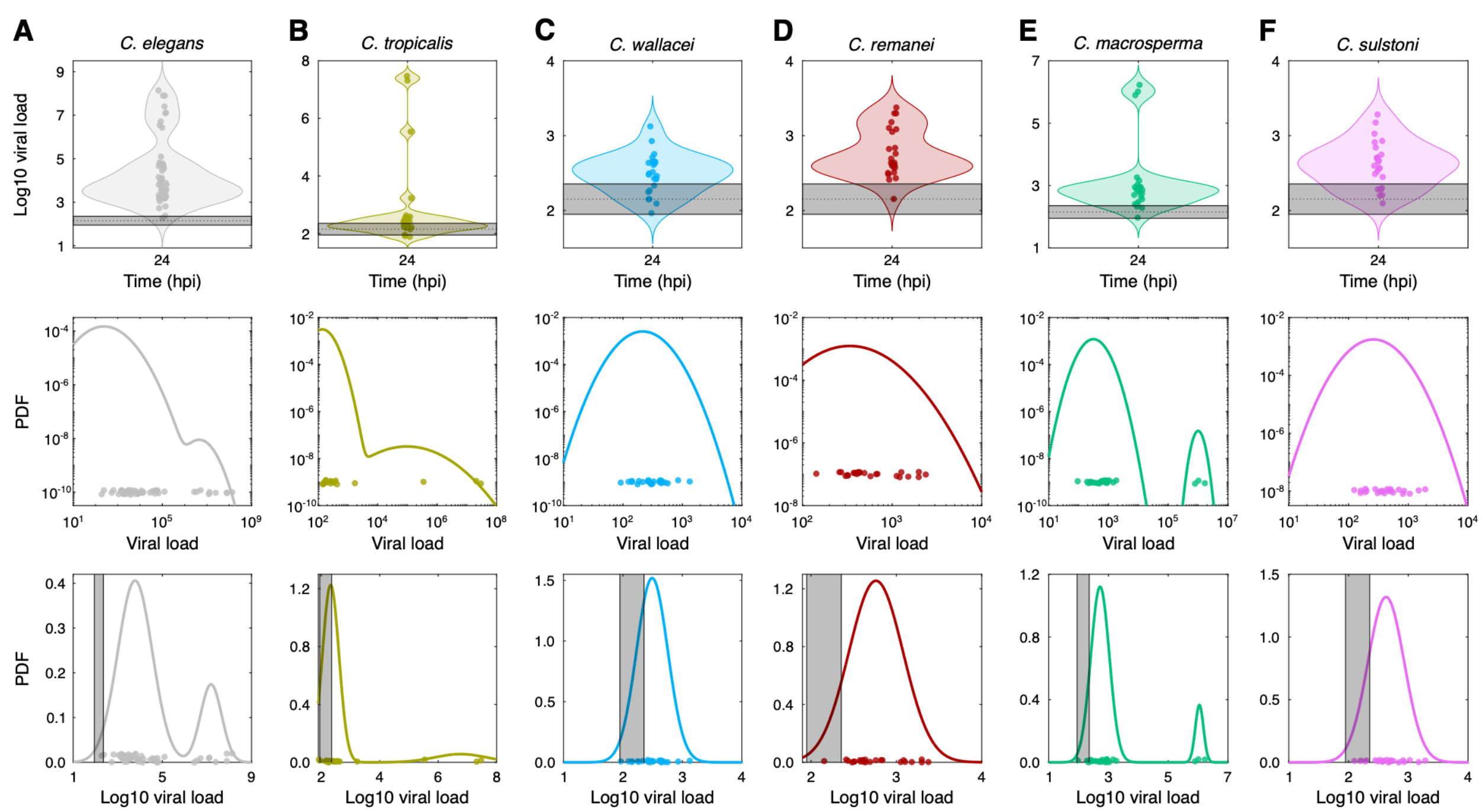

Figure B. Representative images of distinct infection phenotypes observed by smiFISH fluorescence microscopy. *C. elegans* worms at 14 or 24 hpi showing (**A**) infected animals with OrV localized in a cell, in the intestinal lumen, or in both, and (**B**) infected animals with 1, 2, 3, or 4 infected cells. The brightness of each image was adjusted separately.

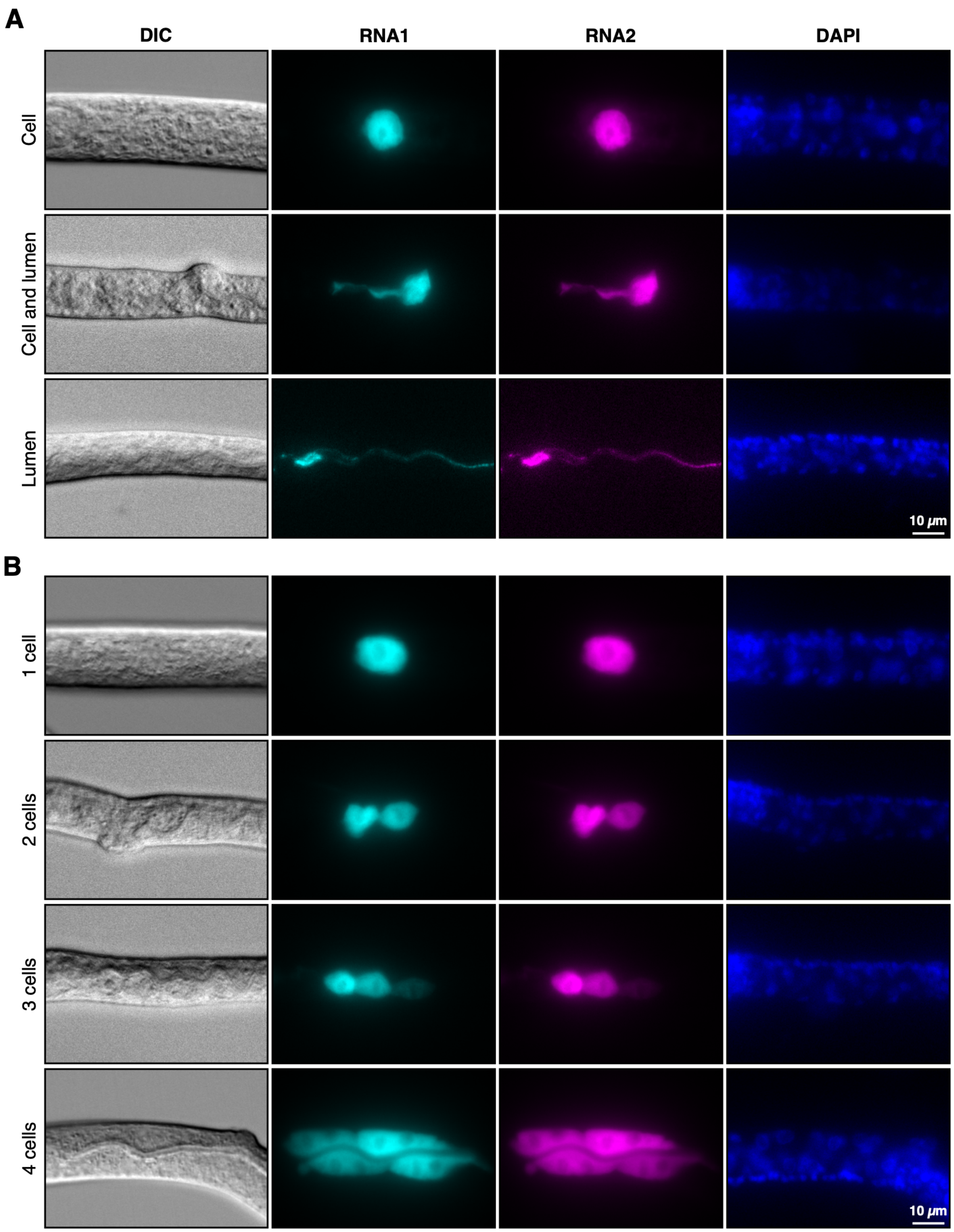

Figure C. Experimental evolution of OrV in *C. elegans* and alternative hosts. (**A**) Experimental design. Five independent lineages of OrV were passaged in each host. Synchronized populations of 20 animals were inoculated with 2.8×10^9 copies of OrV RNA2 to start the first passage. Once starved, plates were washed and evolved virus was filtered out. To inoculate each subsequent passage, 100 µL of virus filtrate from the previous passage was used. There were two experimental regimes that differed in the age of the worms which were inoculated to start a passage, either L1 or L4. (**B**) OrV viral load, measured as number of RNA2 copies/µL, after each passage of experimental evolution starting with L1-inoculated animals. (**C**) OrV viral load, after each passage of experimental evolution starting with L4-inoculated animals. *C. elegans* (grey), *C. tropicalis* (yellow), *C. wallacei* (blue), *C. remanei* (red), *C. macrosperma* (green), and *C. sulstoni* (pink).

The data underlying this Figure can be found in https://doi.org/10.5281/zenodo.19472999 (S3-evolution.xlsx).

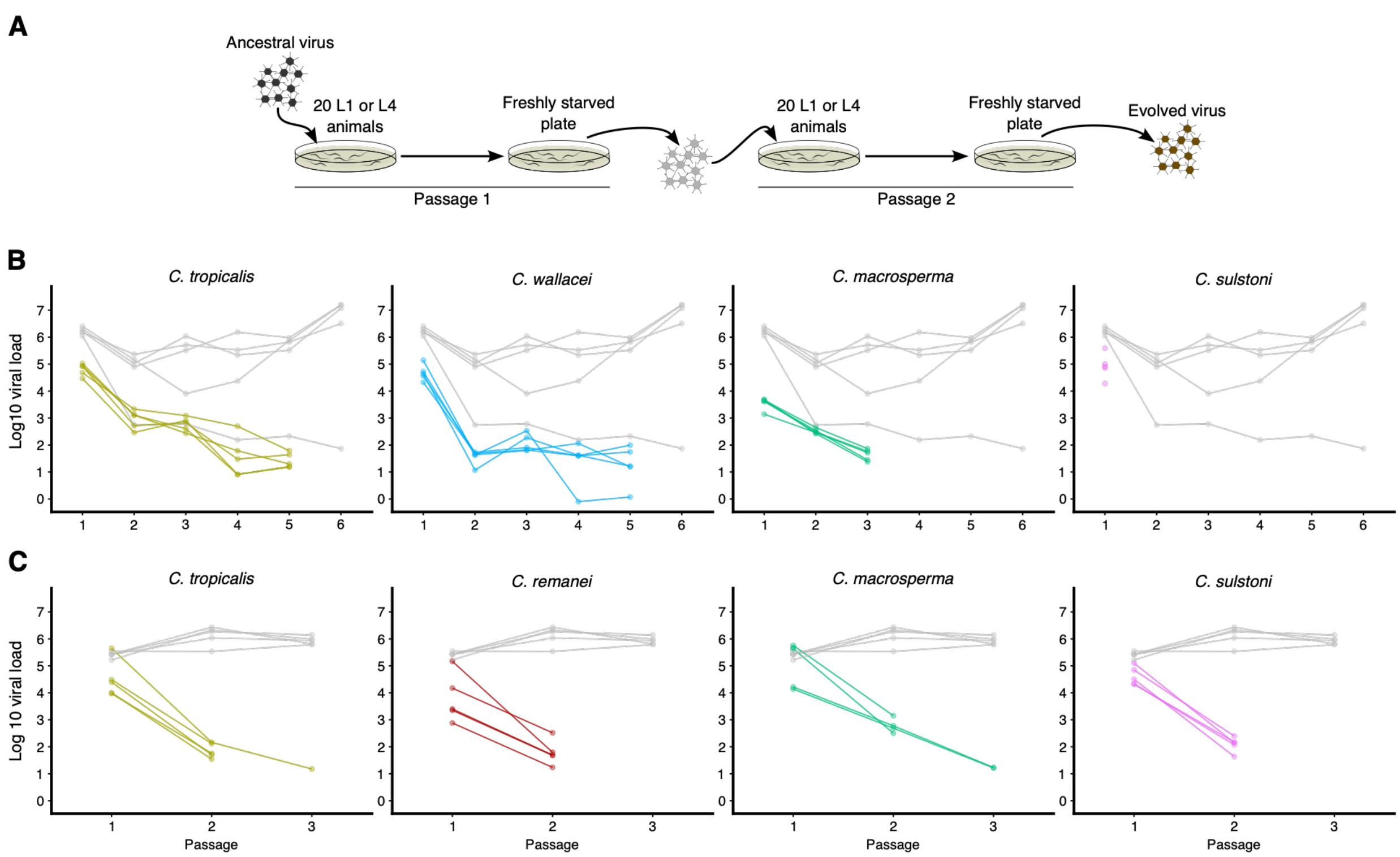

Figure D. Individual worm viral load of *C. elegans* strains ERT54 and N2. OrV viral load of individual animals (n = 24 for each strain) at 24 hpi. Viral load was measured as the number of OrV RNA2 copies in 1/5 of the single worm lysate. Horizontal grey line represents the technical detection limit (mean + 2 SD of negative controls). The viral loads of the two strains do not differ significantly (Kruskal-Wallis test, χ^2 = 0.365, 1 d.f., P = 0.5457).

The data underlying this Figure can be found in https://doi.org/10.5281/zenodo.19472999 (Fig3H_FigS4-individual_viral_load.xlsx).

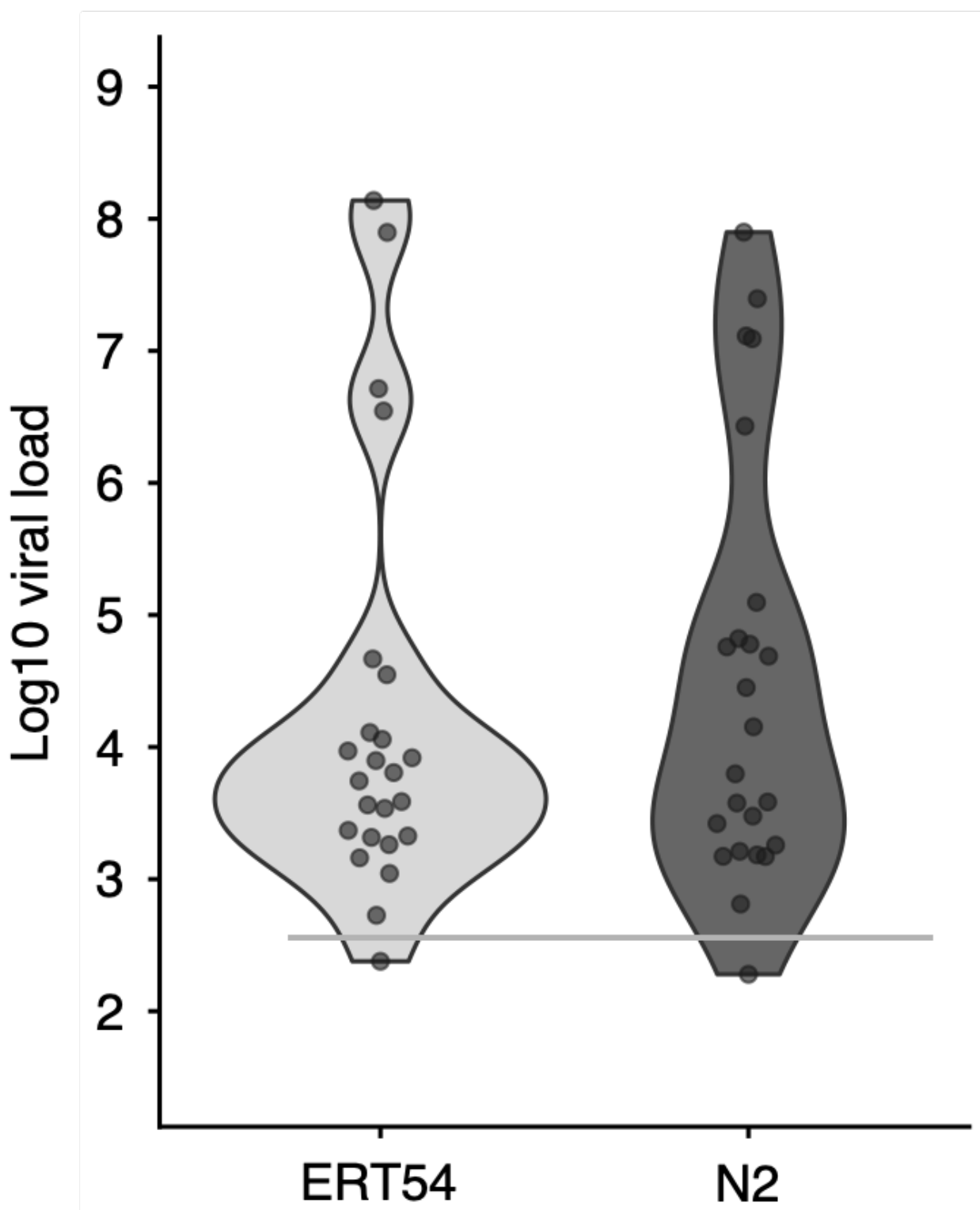

Table A. Regression analysis of $\log_{10}$(RNA2) *vs* $\log_{10}$(RNA1) (Fig. 3C). For each species and time point, the goodness of fit to the corresponding *C. elegans* linear regression model was compared to the fit to its own regression model using BIC evidence weights. In all cases, weights strongly supported species-specific models.

		BIC					
**Species**	**hpi**	***C. elegans* model**	**Own model**	**Evidence weight**	**Intercept (±1 SE)**	**Slope (±1 SE)**	**Adjusted R^2**
C. elegans	14	9.6726			0.1210±0.1811	0.200±0.0403***	0.8063
	24	8.7889			−0.8989±0.0582***	1.0672±0.0582***	0.8072
C. remanei	14	17.8268	6.5547	0.9964	−0.6335±0.2732	0.9621±0.1103**	0.9495
C. tropicalis	24	21.8588	18.3979	0.8485	−9.9848±5.2589	2.6754±0.9412$^{\cdot}$	0.6390
C. wallacei	24	17.9038	−10.5265	1.0000	−3.0002±0.1136*	0.9482±0.0201*	0.9991
C. macrosperma	24	45.6725	36.2643	0.9910	−4.0655±2.2400	1.6710±0.8877	0.2412
C. sulstoni	24	17.5828	−12.7322	1.0000	−3.4314±0.0239**	1.0403±0.0055**	0.9999

Significance levels: $^{\cdot}$ 0.1 > P ≥ 0.05; * 0.05 > P ≥ 0.01; ** 0.01 > P ≥ 0.001; *** 0.001 > P
