# Supplementary material for: Species-specific barriers restrict virus spillover potential across the Caenorhabditis genus
Source: PLoS Biol. 2026 Aug 18;24(8):e3003965. doi: 10.1371/journal.pbio.3003965 (PMC13497278; doi:10.1371/journal.pbio.3003965)
Supplement: Supplementary file 2 [file pbio.3003965.s004.pdf]

Response to the Editor

Summary comments: The editor notes that Reviewer 1 is extremely positive; Reviewer 2 is also impressed but raises concerns about the evidence for the "entry barrier," evidence for completion of the viral life cycle, overly strong conclusions, low sample size for *C. remanei*, and the reliance on only 20 worms for transmission assays. Reviewer 3 is positive and emphasizes improving accessibility by laying out the barriers more clearly, potentially graphically. During cross-commenting, reviewers reached consensus that the manuscript should improve clarity for broad readers, preferably through a graphical representation of the seven barriers and expected patterns. The low sample size issue was also highlighted as a concern.

Response: We thank the editor for this helpful summary. We have revised the manuscript with these priorities in mind. First, we have added a new graphical summary, Fig. 6, that explicitly defines the seven barriers, shows the expected data pattern associated with each one, and summarizes how each *Caenorhabditis* species maps onto the barrier framework.

Second, we have revised several mechanistic interpretations to more clearly distinguish direct observations from inferred mechanisms. In particular, we changed "entry barrier" to "susceptibility, establishment and early-cycle progression"; certainly, the current experiments do not directly separate viral entry from uncoating, early replication, or rapid clearance. We also softened the interpretation of luminal viral RNA, stating that it is consistent with egress or luminal viral accumulation but does not by itself prove the presence of infectious virions.

Third, we added explicit limitations regarding *C. remanei* and the transmission assays. We now state that the *C. remanei* phenotype is informative but should be interpreted cautiously because the number of smiFISH-positive animals is low. We also clarify that the 20-donor transmission assay imposes a standardized exposure regime but may underestimate rare transmission from low-prevalence hosts.

Finally, we revised the experimental evolution section to emphasize that our conclusions apply to the standardized filtration-based passaging protocol used here. We no longer frame the results as demonstrating an absolute inability of OrV to evolve in alternative hosts.

Response to Reviewer #1

We thank Reviewer 1 for the very positive assessment of our work and for the constructive comments. We are particularly grateful for the suggestion to clarify several sections and to perform an additional analysis of the RNA2:RNA1 relationship in Fig. 3C.

Comment 1: Lines 114–115: The Alkan and Felix-Wang references seem out of place here. Previous studies have found variation in susceptibility within native host species, but unless I'm misremembering, only Shaw and Kennedy looked for variable susceptibility in non-native species.

Response 1: We have revised the sentence to distinguish studies documenting natural OrV infection and host-virus diversity from those specifically addressing susceptibility or host range across non-native *Caenorhabditis* species. We now cite Shaw and Kennedy specifically in relation to variable susceptibility and host range in non-native species, while retaining Félix and Wang and Alkan et al. where appropriate for broader background on natural viruses and host-virus specificity.

Comment 2: Lines 206–213: I had trouble following the logic here. The authors are comparing an early timepoint to a later time point, but they use different species to make this comparison. I think all they are trying to say is that remanei infections tended to have undetectable RNA2 expression, whereas the other species did not (and that it might be why Orsay was unable to be detected in a remanei worm at 24 hours). I agree with the plausibility of this idea, but it is worded in a really confusing way.

Response 2: We have rewritten this section to state the result more directly: at 14 hpi, *C. remanei* showed a higher fraction of infected animals/cells in which RNA2 was not detected, whereas this pattern was uncommon in *C. elegans* and in the other species examined at 24 hpi. We now present this as evidence consistent with early-cycle failure or rapid clearance in *C. remanei*, while avoiding an overinterpretation of the exact underlying mechanism.

Comment 3: Line 233 and 244: The authors talk about 'desynchronized' and a temporal shift, but all I see is a lack of RNA2 expression leading to a deficiency in either packaging or egress. Where is the desynchronization?

Response 3: Our use of "desynchronization" was intended to refer to the broader set of observations across the study, including delayed or reduced viral accumulation, altered RNA2:RNA1 ratios, and differences in luminal localization and transmission timing. However, in the specific context flagged by the reviewer, the data more directly support altered RNA2 accumulation and/or impaired progression toward packaging or egress.

We have therefore revised the relevant sentences to avoid invoking “desynchronization” where it is not directly shown. We now reserve the idea of temporal mismatch for sections where it is supported by time-course viral-load data and transmission timing, and we use more precise language in the RNA stoichiometry/localization sections.

Comment 4: Line 290: I'm not sure I understand what ‘transmission model’ means here. I wonder if there is a better term for what is intended.
Response 4: We have replaced this phrase with “relationship between viral accumulation and transmission”. This more accurately describes the observed species-specific differences without implying any formal mathematical model.

Comment 5: Lines 299 and 302: The authors say ‘infected animals’ when I think what they actually mean is ‘animals from infected plates’.
Response 5: It is true that these assays compare animals from inoculated plates with animals from control plates, and not all animals in inoculated populations were necessarily infected. We have replaced “infected animals” with “animals from inoculated plates” or “inoculated animals” throughout this section.

Comment 6: Lines 366–376: This point was also made by Shaw and Kennedy 2025, and might be worth referencing here. This statement is not meant to undercut the novelty of the current study, which is truly excellent. The two studies make a similar statement — there are multiple reasons a host jump can fail — at very different levels of resolution.
Response 6: We now cite Shaw and Kennedy (2025) in this Discussion paragraph, explicitly recognizing that their work also supports the idea that post-spillover persistence depends on multiple epidemiological traits. We then clarify that our contribution is complementary, resolving the underlying mechanistic and cellular barriers that can generate such host-competence differences.

Comment 7: Line 414: ‘Completion of the infection cycle depended strongly on RNA2:RNA1 balance’. This strikes me as an assumption, not a proven fact. Perhaps it could be reworded from ‘depended strongly on’ to ‘associated strongly with’.
Response 7: We have replaced the causal phrase “depended strongly on” with “was strongly associated with”. More generally, we have revised the Discussion to avoid implying that our data directly prove causality between RNA2:RNA1 balance and completion of the viral cycle. We now frame RNA2:RNA1 imbalance as a plausible mechanistic correlate of impaired progression toward transmissible infection.

Comment 8: In Fig 3 panel C. If I understand correctly, the key comparison here is whether the line from C. elegans data can explain the data from the non-native species. To do that the authors could have done model comparison where the 'null model' uses the C. elegans line and the 'full model' is fit to the data. Then a p-value can be generated using a likelihood ratio test that compares these two models.

Response 8: We have performed the suggested model comparison analysis. Specifically, for each non-native species/time-point combination, we compared a model in which the $\log_{10}$RNA2 *vs* $\log_{10}$RNA1 relationship was constrained to the corresponding *C. elegans* relationship with a model in which the slope and/or intercept were allowed to vary for the alternative host. We then evaluated improvement in model fit using BIC evidence weights (*aka* Bayesian weights).

The results of this analysis are now mentioned in the Results and presented in the new Supplementary Table S1. Fig. 3C legend has been updated accordingly. This formal test supports the interpretation that alternative hosts deviate from the *C. elegans* RNA2:RNA1 relationship, although we now phrase this conservatively as altered relative RNA segment accumulation rather than as definitive evidence of failed packaging.

Response to Reviewer #2

We thank Reviewer 2 for the thoughtful and balanced assessment. We appreciate the recognition that the study provides a comprehensive multiscale framework linking infection establishment, replication dynamics, genome-segment balance, transmission, virulence, and evolutionary persistence. We also agree that some mechanistic interpretations in the previous version were too strong relative to the direct evidence.

Comment 1: First, the evidence supporting the proposed 'entry barrier' is limited. While the authors document reduced infection establishment in several host species, the experiments do not directly measure viral entry and therefore cannot distinguish among defects in entry, uncoating, early replication, or rapid viral clearance. Moreover, because smiFISH targets positive-strand viral RNA, the detected signals may include ingested viral particles rather than exclusively replicating virus.

Response 1: We agree that our experiments do not directly measure viral entry, uncoating, or the earliest steps of replication. Our smiFISH assay detects positive-strand viral RNA after inoculation and therefore cannot, by itself, distinguish among entry, uncoating, early replication, host clearance, or other early-cycle processes. For this reason, we have renamed the first barrier from "Susceptibility and entry" to "Susceptibility, establishment, and early-cycle progression," and we now interpret reduced smiFISH positivity as evidence for an early bottleneck in infection establishment or progression, rather than as direct evidence for an entry defect.

At the same time, we have clarified the interpretation of smiFISH positivity to avoid implying that positive signals are likely to reflect merely ingested inoculum. Previous work in the *C. elegans*-OrV system, including smiFISH time-course experiments and comparisons between single-worm viral loads and smiFISH signal, indicates that smiFISH is less sensitive than RT-qPCR and requires relatively high levels of viral RNA accumulation to generate a detectable signal. Under our inoculation protocol, we have not observed positive smiFISH signals attributable solely to ingestion of the viral inoculum. Thus, while smiFISH does not directly demonstrate active replication, detectable signal is most consistent with substantial viral RNA accumulation after exposure.

We have revised the Results and Discussion accordingly. The manuscript now states that smiFISH positivity is interpreted together with viral-load time courses, RNA1/RNA2 co-detection, localization patterns, and transmission assays to infer whether infection progresses beyond early viral RNA detection. This revised wording preserves the reviewer's important caution that smiFISH is not a direct entry or replication assay, while avoiding an overstatement that inoculum ingestion alone is a likely explanation for the observed positive signals.

Comment 2: Second, the interpretation of viral localization within the intestinal lumen as evidence of successful egress and completion of the viral life cycle may be overstated. Lumen-associated viral RNA does not necessarily demonstrate the presence of infectious virions or successful transmission-competent progeny.

Response 2: We have revised the text to state that luminal RNA signal is consistent with viral egress, shedding or luminal accumulation of viral material, but does not by itself demonstrate that infectious virions are present. We now explicitly state that transmission assays provide the functional evidence for infectious output, whereas smiFISH localization provides spatial information that can be interpreted only in combination with transmission data.

This distinction is now also reflected in the graphical barrier framework, where the egress/localization barrier is labeled as "egress/luminal localization" rather than simply "egress."

Comment 3: Third, some conclusions drawn from the experimental evolution studies appear stronger than warranted by the available data. The observed failure of persistence in alternative hosts may indeed reflect evolutionary constraints, but it could also be influenced by characteristics of the passage regime, transmission bottlenecks, or inoculum size.

Response 3: Our serial passages experiment was designed as a standardized test of whether OrV could persist under a filtration-based passaging regime across host species. However, we agree that failure under this regime does not demonstrate an absolute inability of OrV to adapt to alternative hosts. It may also reflect the number of donor animals, bottleneck severity, inoculum dose, infection prevalence, timing of collection, or the fact that filtration-based passaging selects primarily on virus released into the medium.

We now state that alternative hosts functioned as evolutionary sinks under our experimental passaging conditions, rather than as an intrinsic or universal property. We also expanded the Discussion to compare our design with alternative passaging approaches, including worm-picking regimes, which may alter the selective environment by carrying host-associated virus or host traits across passages.

Comment 4: Finally, several mechanistic interpretations rely heavily on observations from C. remanei, where sample sizes are relatively small, and these findings should therefore be presented more cautiously.

Response 4: We agree. *C. remanei* is informative because it shows an unusual phenotype: low detectable viral accumulation at later time points but measurable transmission in some assays. However, because the number of smiFISH-positive *C. remanei* animals is small, mechanistic conclusions must be cautious.

We have revised the relevant Discussion to frame the *C. remanei* pattern as a plausible interpretation rather than a definitive mechanistic conclusion. We now describe *C. remanei* as

suggesting a narrow window of infection progression and potential transmission, but we explicitly state that this hypothesis requires further testing with higher sampling density and direct measures of infectious virion production.

Comment 5: In addition, the transmission assays utilize only 20 worms as infection sources. Given the relatively low levels of viral replication observed in several host species, it is unclear whether this sample size provides sufficient power to accurately assess transmission competence. A discussion of this limitation would strengthen the study.

Response 5: We agree that the 20-donor transmission assay has limitations, particularly for host species in which infection prevalence is low or infection is spatially or behaviorally constrained. Our goal with this assay was to compare transmission under a standardized donor-population regime across host species, rather than to estimate the maximum possible transmission capacity of each species under optimized conditions.

We have now clarified that this population-based assay captures a more ecological, group-level transmission scenario, in which not only viral production but also host behavior can influence exposure of recipient animals. In particular, some species, especially *C. wallacei* and *C. remanei*, tend to aggregate into one or two groups on the plate and remain there for much of the 24 h transmission window. Such aggregation may reduce movement across the plate and limit contact with healthy *C. elegans* ERT54 recipients, thereby lowering apparent transmission in the population-donor assay even if some individual animals are capable of producing transmissible virus.

We also now emphasize that the 20-donor assay is complemented by the single-worm transmission assay. The latter is less influenced by the uncertainty over how many infected animals are present in a donor group and is better suited to asking whether individual animals can produce infectious virus capable of initiating infection in recipient *C. elegans*. This distinction is particularly relevant for *C. remanei*, where population-level transmission was low but individual transmission was comparatively high, suggesting that differences between the two assays may reflect behavioral and contact-structure effects in addition to viral production.

Comment 6: Overall, this is a well-executed and biologically meaningful study … However, several mechanistic interpretations extend beyond the direct evidence presented and would benefit from more cautious wording."

Response 6: We thank the reviewer for this constructive summary and have revised the manuscript throughout to better distinguish observations from interpretations. The main edits include:

- replacing "entry barrier" with "early establishment/early-cycle progression";

- replacing “successful egress” with “luminal localization consistent with egress or viral material accumulation”;
- replacing “completion of the viral cycle” with “progression toward a potentially transmissible stage”;
- replacing “depended on RNA2:RNA1 balance” with “was associated with RNA2:RNA1 balance”;
- revising experimental-evolution conclusions to apply specifically to our passaging regime;
- adding limitations regarding *C. remanei* sample size and donor number in transmission assays.

We believe these revisions preserve the central conclusion while making the mechanistic claims more proportionate to the evidence.

Minor point 1. Line 244: ‘delays or prevent egression’ — > ‘delays or prevents egress’.
Response: This sentence already changed.
Minor point 2. Line 323: ‘20 synchronized worms who were left to grow’ — > ‘20 synchronized worms that were left to grow’.
Response: This paragraph has been fully changed.

Response to Reviewer #3

We thank Reviewer 3 for the positive assessment and for emphasizing accessibility. We agree that the manuscript contains many species, assays, and barriers, and that the barrier framework needed to be made more explicit for readers. We have therefore revised the manuscript to define, visualize, and summarize the seven barriers more clearly.

Comment 1: To reach its full potential, I think that it's essential to explain and visualize your verbal model of the different barriers. As there are necessarily a lot of results — 6 species and 7 barriers — it would be very helpful to clearly layout the expectations for each barrier. This would also allow you to come back to these expectations, and easily graphically summarize the results by barrier and species. As it stands, the results are hard to follow, and the conclusion in the result is overly general."

Response 1: We have added a new graphical summary, Fig. 6, that explicitly lays out the seven barriers, the measurement associated with each barrier, and the expected pattern if that barrier is limiting. The figure also summarizes the qualitative outcome for each host species, allowing readers to see how the different barriers combine to produce distinct host-competence phenotypes.

We have also revised the Results to introduce the barrier framework more clearly before moving into the individual assays. At the end of the Results, we now return to the framework and summarize how each species maps onto the seven barriers. This makes the final Results conclusion more specific and less general.

Comment 2: I don't know the practicalities of the nematode assays well enough, and I failed to gather it from the methods; but if multiple individuals were maintained in one plate, and you used multiple plates, then plate should be a random factor … If so, I would still suggest you clarify the setup a bit more, so that it becomes clear that it doesn't require more complicated models.

Response 2: We have clarified the experimental setup in the Methods. In our design, biological replicates corresponded to independently inoculated populations/plates, and the statistical unit was the replicate population or independently sampled individual, depending on the assay. For assays in which multiple animals were measured from the same population, we have clarified how replicate structure was handled statistically.

Where appropriate, we have revised the Methods to state whether plate identity was equivalent to biological replicate, whether measurements were averaged at the replicate level, or whether replicate/plate was included as a factor. We have also checked that the conclusions are not driven by pseudoreplication.

Comment 3: To improve readability: Explain abbreviations (hpi, smiFISH…) in results.

Response 3: Done. We also checked the manuscript for other abbreviations that may be unfamiliar to a broad readership.
