# Supplementary material for: Species-specific barriers restrict virus spillover potential across the Caenorhabditis genus
Source: PLoS Biol. 2026 Aug 18;24(8):e3003965. doi: 10.1371/journal.pbio.3003965 (PMC13497278; doi:10.1371/journal.pbio.3003965)
Supplement: Supplementary file 3 [file pbio.3003965.s005.pdf]

Responses to Academic Editor:

Question: a) Please change your Title to ""Species-specific barriers restrict virus spillover potential across the Caenorhabditis genus" - we think this better reflects this implications for understanding barriers to spillover; the name of the virus is clearly apparent in the Abstract for those who wish.

Answer: Done. Thank you for the suggestion.

Question: b) The Academic Editor had some presentational suggestions, which we think are a good idea, and may better address what reviewer #3 was looking for. Specifically, the AE said, "I have a suggestion though, regarding the first point of Reviewer #3. The reviewer recommends a concept figure with the barrier model and a summary figure in the end, referring to the concept figure. In response, the authors have introduced a two-part Figure 6 with a concept part A and a results summary in B. They also say: 'We have also revised the Results to introduce the barrier framework more clearly before moving into the individual assays.' I suggest to split Fig 6 and introduce the concept in the beginning of the Results. By the way, I do not see a substantially clearer introduction of the barrier framework in the revised Results. The general sentence in the beginning of the Results is edited only mildly, and then they go pretty straight into the smiFISH assay as before. An early introduction of the barrier framework would make the paper clearer in my view." Please address these points.

Answer: Thank you for the clear suggestion. Following your advice, we have split the original Fig. 6 in two. New Fig. 1 is the concept figure and is presented at the beginning of the Results section. New Fig. 7 is the summary figure, now first presented at the end of the Results section and linking with the conceptual model.

Furthermore, we have rewritten the first paragraph of the Results to clearly introduce the barriers-resolved model.

In addition, we have rewritten the first three paragraphs of the Discussion to highlight the barriers model, citing Figs. 1 and 7.

Question: c) Please address my Data Policy requests below; specifically, we need you to supply the numerical values underlying Figs 1C, 2BCDEFGH, 3ABCDEF, 4BC, 5ABCDEF, S1ABCDEF, S3BC, S4, either as a supplementary data file or as a permanent DOI'd deposition. I note that you already have an associated Zenodo deposition (https://urldefense.com/v3/__https://doi.org/10.5281/zenodo.19473000__;!!D9dNQwwGXtA!SQVXCCdw122dQ5xt5kCbLxmjd-mn1-b1BDNXuA9cCEHb_nFSkHbH4ozl43iQFXddzgCchkzsHA2qNXhEKHQ$), and that this explicitly contains data underlying most of these Figures; however, I couldn't find data underlying Figs S1ABCDEF and S3BC; please could you supply this and/or clarify?

Answer: We have uploaded into Zenodo the dataset underlying Fig. S3.

Please, notice that data underlying Fig. S1A-F are the same that for (now) Fig. 3H. This is now clear with the addition of the Zenodo links into the figure legends.

Question: d) Please cite the location of the data clearly in all relevant main and supplementary Figure legends, e.g. "The data underlying this Figure can be found in S1 Data" or "The data underlying this Figure can be found in https://urldefense.com/v3/__https://zenodo.org/records/19473000__;!!D9dNQwwGXtA!SQVXCCdw122dQ5xt5kCbLxmjd-mn1-b1BDNXuA9cCEHb_nFSkHbH4ozl43iQFXddzgCchkzsHA2qY4GSkIU$ "; also update your Data Availability Statement accordingly.

Answer: Following the instructions, we have added Zenodo links to the datasets in the figure legends, with specific indication of the corresponding datafiles underlying the figures.

Question: e) Please include the URLs of your funders in the Financial Disclosure statement. **Answer**: Done.
