# Supplementary material for: Species-specific barriers restrict virus spillover potential across the Caenorhabditis genus
Source: PLoS Biol. 2026 Aug 18;24(8):e3003965. doi: 10.1371/journal.pbio.3003965 (PMC13497278; doi:10.1371/journal.pbio.3003965)
Supplement: Peer review history

# Editor report: Decision Letter 0

**DOI:** 10.1371/journal.pbio.3003965.r001

**Authors:** Roland G Roberts

23 May 2026

Dear Dr Elena,

Thank you for submitting your manuscript entitled "Species-specific barriers constrict Orsay virus host range across the Caenorhabditis genus" for consideration as a Research Article by PLOS Biology.

Your manuscript has now been evaluated by the PLOS Biology editorial staff, as well as by an academic editor with relevant expertise, and I'm writing to let you know that we would like to send your submission out for external peer review.

However, before we can send your manuscript to reviewers, we need you to complete your submission by providing the metadata that is required for full assessment. To this end, please login to Editorial Manager where you will find the paper in the 'Submissions Needing Revisions' folder on your homepage. Please click 'Revise Submission' from the Action Links and complete all additional questions in the submission questionnaire.

Once your full submission is complete, your paper will undergo a series of checks in preparation for peer review. After your manuscript has passed the checks it will be sent out for review. To provide the metadata for your submission, please Login to Editorial Manager (https://www.editorialmanager.com/pbiology) within two working days, i.e. by May 27 2026 11:59PM.

If your manuscript has been previously peer-reviewed at another journal, PLOS Biology is willing to work with those reviews in order to avoid re-starting the process. Submission of the previous reviews is entirely optional and our ability to use them effectively will depend on the willingness of the previous journal to confirm the content of the reports and share the reviewer identities. Please note that we reserve the right to invite additional reviewers if we consider that additional/independent reviewers are needed, although we aim to avoid this as far as possible. In our experience, working with previous reviews does save time.

If you would like us to consider previous reviewer reports, please edit your cover letter to let us know and include the name of the journal where the work was previously considered and the manuscript ID it was given. In addition, please upload a response to the reviews as a 'Prior Peer Review' file type, which should include the reports in full and a point-by-point reply detailing how you have or plan to address the reviewers' concerns.

During the process of completing your manuscript submission, you will be invited to opt-in to posting your pre-review manuscript as a bioRxiv preprint. Visit http://journals.plos.org/plosbiology/s/preprints for full details. If you consent to posting your current manuscript as a preprint, please upload a single Preprint PDF.

Feel free to email us at plosbiology@plos.org if you have any queries relating to your submission.

Kind regards,

Roli Roberts

Roland Roberts, PhD

Senior Editor

PLOS Biology

rroberts@plos.org

# Peer review: Decision Letter 1

**DOI:** 10.1371/journal.pbio.3003965.r002

**Authors:** Roland G Roberts

10 Jul 2026

Dear Dr Elena,

Thank you for your patience while your manuscript "Species-specific barriers constrict Orsay virus host range across the Caenorhabditis genus" went through peer-review at PLOS Biology. Your manuscript has now been evaluated by the PLOS Biology editors, an Academic Editor with relevant expertise, and by three independent reviewers.

You'll see that reviewer #1 is extremely positive; most of his/her requests are textual though s/he ends with a point that requires a minor analysis. Reviewer #2 is also impressed, but raises a number of concerns. These involve the evidence for the “entry barrier,” the evidence for completion of the viral life cycle, some excessively strong conclusions, the low sample size for C. remanei, and the reliance on only 20 worms for transmission assays. Reviewer #3 is also very positive, and his/her main concern is the accessibility of the manuscript; s/he suggests that you lay out the barriers more clearly, and potentially graphically (she also has a methodological query).

During the cross-commenting phase, a strong consensus emerged for improving the clarity of the manuscript for the broader reader, preferably by some sort of graphical representation (one reviewer said, "I also really liked the suggestion of reviewer 3 to include a figure that explicitly lays out the '7 barriers' and the expected patterns that would be seen in the data if each barrier were the limiting one"; another said, "each section should more clearly describe the experiment associated with it and it could be illustrated in a figure"). The low sample size issue, flagged by reviewer #2, is also a concern.

In light of the reviews, which you will find at the end of this email, we are pleased to offer you the opportunity to address the comments from the reviewers in a revision that we anticipate should not take you very long. We will then assess your revised manuscript and your response to the reviewers' comments with our Academic Editor aiming to avoid further rounds of peer-review, although we might need to consult with the reviewers, depending on the nature of the revisions.

In addition to these revisions, you may need to complete some formatting changes, which you will receive in a follow up email. A member of our team will be in touch with a set of requests shortly. If you do not receive a separate email within a few days, please assume that checks have been completed, and no additional changes are required.

We expect to receive your revised manuscript within 1 month. Please email us (plosbiology@plos.org) if you have any questions or concerns, or would like to request an extension.

At this stage, your manuscript remains formally under active consideration at our journal; please notify us by email if you do not intend to submit a revision so that we withdraw the manuscript.

**IMPORTANT - SUBMITTING YOUR REVISION**

Your revisions should address the specific points made by each reviewer. Please submit the following files along with your revised manuscript:

1. A 'Response to Reviewers' file - this should detail your responses to the editorial requests, present a point-by-point response to all of the reviewers' comments, and indicate the changes made to the manuscript.

*NOTE: In your point-by-point response to the reviewers, please provide the full context of each review. Do not selectively quote paragraphs or sentences to reply to. The entire set of reviewer comments should be present in full and each specific point should be responded to individually.

You should also cite any additional relevant literature that has been published since the original submission and mention any additional citations in your response.

2. In addition to a clean copy of the manuscript, please also upload a 'track-changes' version of your manuscript that specifies the edits made. This should be uploaded as a "Revised Article with Changes Highlighted " file type.

*Resubmission Checklist*

When you are ready to resubmit your revised manuscript, please refer to this resubmission checklist: https://plos.io/Biology_Checklist

To submit a revised version of your manuscript, please go to https://www.editorialmanager.com/pbiology/ and log in as an Author. Click the link labelled 'Submissions Needing Revision' where you will find your submission record.

Please make sure to read the following important policies and guidelines while preparing your revision:

*Published Peer Review*

Please note while forming your response, if your article is accepted, you may have the opportunity to make the peer review history publicly available. The record will include editor decision letters (with reviews) and your responses to reviewer comments. If eligible, we will contact you to opt in or out. Please see here for more details:

https://blogs.plos.org/plos/2019/05/plos-journals-now-open-for-published-peer-review/

*PLOS Data Policy*

Please note that as a condition of publication PLOS' data policy (http://journals.plos.org/plosbiology/s/data-availability) requires that you make available all data used to draw the conclusions arrived at in your manuscript. If you have not already done so, you must include any data used in your manuscript either in appropriate repositories, within the body of the manuscript, or as supporting information (N.B. this includes any numerical values that were used to generate graphs, histograms etc.). For an example see here: http://www.plosbiology.org/article/info%3Adoi%2F10.1371%2Fjournal.pbio.1001908#s5

*Blot and Gel Data Policy*

We require the original, uncropped and minimally adjusted images supporting all blot and gel results reported in an article's figures or Supporting Information files. We will require these files before a manuscript can be accepted so please prepare them now, if you have not already uploaded them. Please carefully read our guidelines for how to prepare and upload this data: https://journals.plos.org/plosbiology/s/figures#loc-blot-and-gel-reporting-requirements

*Protocols deposition*

To enhance the reproducibility of your results, we recommend that if applicable you deposit your laboratory protocols in protocols.io, where a protocol can be assigned its own identifier (DOI) such that it can be cited independently in the future. Additionally, PLOS ONE offers an option for publishing peer-reviewed Lab Protocol articles, which describe protocols hosted on protocols.io. Read more information on sharing protocols at https://plos.org/protocols?utm_medium=editorial-email&utm_source=authorletters&utm_campaign=protocols

Thank you again for your submission to our journal. We hope that our editorial process has been constructive thus far, and we welcome your feedback at any time. Please don't hesitate to contact us if you have any questions or comments.

Sincerely,

Roland

Roland Roberts, PhD

Senior Editor

PLOS Biology

rroberts@plos.org

----------------------------------------------------------------

REVIEWERS' COMMENTS:

Reviewer #1:

The manuscript by Herek and colleagues explores barriers to viral host jumps. To explore this topic, they take a virus of C. elegans and compare the within-host virus dynamics in that host (the native host) to the dynamics in five non-native host species. They find that the virus is unable to sustainably persist in any of the non-native hosts, but that the reasons why differ between hosts - some hosts had low virus accumulation, some hosts had low viral egress, some hosts showed unbalanced production of RNA1 and RNA2. Their ultimate conclusion is that the barriers to host jumps differ between species. I loved this manuscript. It was a super fun and super easy read. All of my comments are fairly minor.

-Lines 114-115: The Alkan and FelixWang references seem out of place here. Previous studies have found variation in susceptibility within native host species, but unless I'm misremembering, only Shaw and Kennedy looked for variable susceptibility in non-native species.

-Lines 206-213: I had trouble following the logic here. The authors are comparing an early timepoint to a later time point, but they use different species to make this comparison. I think all they are trying to say is that remanei infections tended to have undetectable RNA2 expression, whereas the other species did not (and that it might be why Orsay was unable to be detected in a remanei worm at 24 hours). I agree with the plausibility of this idea, but it is worded in a really confusing way.

-Line 233 and 244: The authors talk about "desynchronized" and a temporal shift, but all I see is a lack of RNA2 expression leading to a deficiency in either packaging or egress. Where is the desynchronization?

-Line 290: I'm not sure I understand what "transmission model" means here. I wonder if there is a better term for what is intended.

-Lines 299 and 302: The authors say "infected animals" when I think what they actually mean is "animals from infected plates".

-Lines 366-376: This point was also made by Shaw and Kennedy 2025, and might be worth referencing here. This statement is not meant to undercut the novelty of the current study, which is truly excellent. The two studies make a similar statement (there are multiple reasons a host jump can fail) at very different levels of resolution.

-Line 414: "Completion of the infection cycle depended strongly on RNA2:RNA1 balance". This strikes me as an assumption, not a proven fact. Perhaps it could be reworded from "depended strongly on" to "associated strongly with".

-In Fig 3 panel C. If I understand correctly, the key comparison here is whether the line from C. elegans data can explain the data from the non-native species. To do that the authors could have done model comparison where the "null model" uses the C. elegans line and the "full model" is fit to the data. Then a p-value can be generated using a likelihood ratio test that compares these two models.

Reviewer #2:

This study addresses an important question in virus emergence biology: why some cross-species infections fail despite successful infection and replication. Using the Orsay virus-Caenorhabditis system, the authors combine viral kinetics, smiFISH imaging, transmission assays, virulence measurements, and experimental evolution across six Caenorhabditis species to develop a multiscale view of host competence. The breadth of the experimental design is impressive and provides a valuable opportunity to connect within-host viral processes with transmission dynamics and long-term evolutionary persistence.

The manuscript has several notable strengths. The authors systematically examine multiple stages of the infection process, including susceptibility, viral replication, genome-segment stoichiometry, egress, transmission, virulence, and evolutionary sustainability. This comprehensive approach creates a coherent framework linking molecular and cellular events to population-level outcomes. The conclusion that viral load alone is insufficient to predict transmission is supported by several independent observations across host species, collectively demonstrating that host competence is a multidimensional trait rather than a simple function of viral accumulation. Furthermore, the inclusion of transmission assays and serial-passage experiments substantially increases the biological relevance of the study, as successful emergence ultimately depends on transmission and persistence rather than replication alone. The single-animal analyses are particularly informative, revealing substantial heterogeneity in infection outcomes that would be obscured by population averages.

Although many individual components of the study, including cross-species infection, transmission barriers, and experimental evolution in alternative hosts, have been explored previously, the manuscript's primary innovation lies in its integration of these processes within a single experimental framework. By simultaneously evaluating infection establishment, replication dynamics, genome-segment balance, transmission efficiency, virulence, and evolutionary persistence across multiple host species, the study provides one of the most comprehensive assessments of host competence yet performed in the Orsay virus system. The work represents an important synthesis and extension of existing concepts. Its significance derives from the rigor and scope of the experimental approach and from the insights gained into how multiple, partially independent barriers collectively shape viral host-range expansion and emergence.

Despite these strengths, several concerns should be addressed. First, the evidence supporting the proposed "entry barrier" is limited. While the authors document reduced infection establishment in several host species, the experiments do not directly measure viral entry and therefore cannot distinguish among defects in entry, uncoating, early replication, or rapid viral clearance. Moreover, because smiFISH targets positive-strand viral RNA, the detected signals may include ingested viral particles rather than exclusively replicating virus. Second, the interpretation of viral localization within the intestinal lumen as evidence of successful egress and completion of the viral life cycle may be overstated. Lumen-associated viral RNA does not necessarily demonstrate the presence of infectious virions or successful transmission-competent progeny. Third, some conclusions drawn from the experimental evolution studies appear stronger than warranted by the available data. The observed failure of persistence in alternative hosts may indeed reflect evolutionary constraints, but it could also be influenced by characteristics of the passage regime, transmission bottlenecks, or inoculum size. Finally, several mechanistic interpretations rely heavily on observations from C. remanei, where sample sizes are relatively small, and these findings should therefore be presented more cautiously.

In addition, the transmission assays utilize only 20 worms as infection sources. Given the relatively low levels of viral replication observed in several host species, it is unclear whether this sample size provides sufficient power to accurately assess transmission competence. A discussion of this limitation would strengthen the study.

Overall, this is a well-executed and biologically meaningful study that provides an comprehensive examination of host competence in a natural virus-host system. The central conclusion, that successful viral emergence is shaped by multiple partially independent barriers rather than viral replication alone, is generally well supported by the data. However, several mechanistic interpretations extend beyond the direct evidence presented and would benefit from more cautious wording. Addressing these concerns would further strengthen the manuscript and improve its impact. While the novelty is primarily incremental rather than transformative, the breadth of the experimental framework and the integration of multiple biological scales make this work a valuable contribution to the field.

Minor points

The manuscript is generally well written, but several recurring issues remain.

Line 244: "delays or prevent egression" -- > "delays or prevents egress."

Line 323: "20 synchronized worms who were left to grow" -- > "20 synchronized worms that were left to grow"

Reviewer #3:

This is a very interesting and relevant paper, experimentally testing barriers to disease emergence in a tractable system. The introduction is clear, and the data is clearly interpreted. I really enjoyed reading it, and look forward to this work being published! However, I have some recommendations which I hope will make this manuscript even stronger.

To reach its full potential, I think that it's essential to explain and visualize your verbal model of the different barriers. As there are necessarily a lot of results - 6 species and 7 barriers - it would be very helpful to clearly layout the expectations for each barrier. This would also allow you to come back to these expectations, and easily graphically summarize the results by barrier and species. As it stands, the results are hard to follow, and the conclusion in the result is overly general.

I don't know the practicalities of the nematode assays well enough, and I failed to gather it from the methods; but if multiple individuals were maintained in one plate, and you used multiple plates, then plate should be a random factor (which may also mean that models converge better or that residual environmental variation might be absorbed better). That might not at all be applicable, depending on how the experiments were set up, but e.g. for Drosophila, this would certainly be an issue. If so, I would still suggest you clarify the setup a bit more, so that it becomes clear that it doesn't require more complicated models.

To improve readability: Explain abbreviations (hpi, smiFISH…) in results

# Author comment: Author response to Decision Letter 2

**DOI:** 10.1371/journal.pbio.3003965.r003

22 Jul 2026

Attachment Submitted filename: Rebuttals.pdf

# Editor report: Decision Letter 2

**DOI:** 10.1371/journal.pbio.3003965.r004

**Authors:** Roland G Roberts

3 Aug 2026

Dear Dr Elena,

Thank you for your patience while we considered your revised manuscript "Species-specific barriers constrict Orsay virus host range across the Caenorhabditis genus" for publication as a Research Article at PLOS Biology. This revised version of your manuscript has been evaluated by the PLOS Biology editors and the Academic Editor.

Based on our Academic Editor's assessment of your revision, we are likely to accept this manuscript for publication, provided you satisfactorily address the following data and other policy-related requests.

IMPORTANT - please attend to the following:

a) Please change your Title to ""Species-specific barriers restrict virus spillover potential across the Caenorhabditis genus" - we think this better reflects this implications for understanding barriers to spillover; the name of the virus is clearly apparent in the Abstract for those who wish.

b) The Academic Editor had some presentational suggestions, which we think are a good idea, and may better address what reviewer #3 was looking for. Specifically, the AE said, "I have a suggestion though, regarding the first point of Reviewer #3. The reviewer recommends a concept figure with the barrier model and a summary figure in the end, referring to the concept figure. In response, the authors have introduced a two-part Figure 6 with a concept part A and a results summary in B. They also say: 'We have also revised the Results to introduce the barrier framework more clearly before moving into the individual assays.' I suggest to split Fig 6 and introduce the concept in the beginning of the Results. By the way, I do not see a substantially clearer introduction of the barrier framework in the revised Results. The general sentence in the beginning of the Results is edited only mildly, and then they go pretty straight into the smiFISH assay as before. An early introduction of the barrier framework would make the paper clearer in my view." Please address these points.

c) Please address my Data Policy requests below; specifically, we need you to supply the numerical values underlying Figs 1C, 2BCDEFGH, 3ABCDEF, 4BC, 5ABCDEF, S1ABCDEF, S3BC, S4, either as a supplementary data file or as a permanent DOI’d deposition. I note that you already have an associated Zenodo deposition (https://doi.org/10.5281/zenodo.19473000), and that this explicitly contains data underlying most of these Figures; however, I couldn't find data underlying Figs S1ABCDEF and S3BC; please could you supply this and/or clarify?

d) Please cite the location of the data clearly in all relevant main and supplementary Figure legends, e.g. “The data underlying this Figure can be found in S1 Data” or “The data underlying this Figure can be found in https://zenodo.org/records/19473000"; also update your Data Availability Statement accordingly.

e) Please include the URLs of your funders in the Financial Disclosure statement.

As you address these items, please take this last chance to review your reference list to ensure that it is complete and correct. If you have cited papers that have been retracted, please include the rationale for doing so in the manuscript text, or remove these references and replace them with relevant current references. Any changes to the reference list should be mentioned in the cover letter that accompanies your revised manuscript.

In addition to these revisions, you may need to complete some formatting changes, which you will receive in a follow up email. A member of our team will be in touch with a set of requests shortly. If you do not receive a separate email within a few days, please assume that checks have been completed, and no additional changes are required.

We expect to receive your revised manuscript within two weeks.

To submit your revision, please go to https://www.editorialmanager.com/pbiology/ and log in as an Author. Click the link labelled 'Submissions Needing Revision' to find your submission record. Your revised submission must include the following:

- a cover letter that should detail your responses to any editorial requests, if applicable, and whether changes have been made to the reference list

- a Response to Reviewers file that provides a detailed response to the reviewers' comments (if applicable, if not applicable please do not delete your existing 'Response to Reviewers' file.)

- a track-changes file indicating any changes that you have made to the manuscript.

NOTE: If Supporting Information files are included with your article, note that these are not copyedited and will be published as they are submitted. Please ensure that these files are legible and of high quality (at least 300 dpi) in an easily accessible file format. For this reason, please be aware that any references listed in an SI file will not be indexed. For more information, see our Supporting Information guidelines:

https://journals.plos.org/plosbiology/s/supporting-information

*Published Peer Review History*

Please note that you may have the opportunity to make the peer review history publicly available. The record will include editor decision letters (with reviews) and your responses to reviewer comments. If eligible, we will contact you to opt in or out. Please see here for more details:

https://plos.org/published-peer-review-history/

*Press*

Should you, your institution's press office or the journal office choose to press release your paper, please ensure you have opted out of Early Article Posting on the submission form. We ask that you notify us as soon as possible if you or your institution is planning to press release the article.

*Protocols deposition*

To enhance the reproducibility of your results, we recommend that if applicable you deposit your laboratory protocols in protocols.io, where a protocol can be assigned its own identifier (DOI) such that it can be cited independently in the future. Additionally, PLOS ONE offers an option for publishing peer-reviewed Lab Protocol articles, which describe protocols hosted on protocols.io. Read more information on sharing protocols at https://plos.org/protocols?utm_medium=editorial-email&utm_source=authorletters&utm_campaign=protocols

Please do not hesitate to contact me should you have any questions.

Sincerely,

Roli Roberts

Roland Roberts, PhD

Senior Editor

rroberts@plos.org

PLOS Biology

------------------------------------------------------------------------

DATA POLICY:

You may be aware of the PLOS Data Policy, which requires that all data be made available without restriction: http://journals.plos.org/plosbiology/s/data-availability. For more information, please also see this editorial: http://dx.doi.org/10.1371/journal.pbio.1001797

Note that we do not require all raw data. Rather, we ask that all individual quantitative observations that underlie the data summarized in the figures and results of your paper be made available in one of the following forms:

1) Supplementary files (e.g., excel). Please ensure that all data files are uploaded as 'Supporting Information' and are invariably referred to (in the manuscript, figure legends, and the Description field when uploading your files) using the following format verbatim: S1 Data, S2 Data, etc. Multiple panels of a single or even several figures can be included as multiple sheets in one excel file that is saved using exactly the following convention: S1_Data.xlsx (using an underscore).

2) Deposition in a publicly available repository. Please also provide the accession code or a reviewer link so that we may view your data before publication.

Regardless of the method selected, please ensure that you provide the individual numerical values that underlie the summary data displayed in the following figure panels as they are essential for readers to assess your analysis and to reproduce it: Figs 1C, 2BCDEFGH, 3ABCDEF, 4BC, 5ABCDEF, S1ABCDEF, S3BC, S4 (as mentioned above, we think that most of these are already in your Zenodo depostion, but data for Figs S1ABCDEF and S3BC seem to be missing. NOTE: the numerical data provided should include all replicates AND the way in which the plotted mean and errors were derived (it should not present only the mean/average values).

IMPORTANT: Please also ensure that figure legends in your manuscript include information on where the underlying data can be found, and ensure your supplemental data file/s has a legend.

Please ensure that your Data Statement in the submission system accurately describes where your data can be found.

------------------------------------------------------------------------

CODE POLICY

Per journal policy, if you have generated any custom code during the course of this investigation, please make it available without restrictions. Please ensure that the code is sufficiently well documented and reusable, and that your Data Statement in the Editorial Manager submission system accurately describes where your code can be found. More information on our Code Policy, what and how to share can be found here: https://journals.plos.org/plosbiology/s/code-availability

Please note that we cannot accept sole deposition of code in GitHub, as this could be changed after publication. However, you can archive this version of your publicly available GitHub code to Zenodo. Once you do this, it will generate a DOI number, which you will need to provide in the Data Accessibility Statement (you are welcome to also provide the GitHub access information). See the process for doing this here: https://docs.github.com/en/repositories/archiving-a-github-repository/referencing-and-citing-content

------------------------------------------------------------------------

DATA NOT SHOWN?

- Please note that per journal policy, we do not allow the mention of "data not shown", "personal communication", "manuscript in preparation" or other references to data that is not publicly available or contained within this manuscript. Please either remove mention of these data or provide figures presenting the results and the data underlying the figure(s).

------------------------------------------------------------------------

# Author comment: Author response to Decision Letter 3

**DOI:** 10.1371/journal.pbio.3003965.r005

5 Aug 2026

Attachment Submitted filename: Rebuttal letter.pdf

# Editor report: Decision Letter 3

**DOI:** 10.1371/journal.pbio.3003965.r006

**Authors:** Roland G Roberts

6 Aug 2026

Dear Dr Elena,

Thank you for the submission of your revised Research Article "Species-specific barriers restrict virus spillover potential across the Caenorhabditis genus" for publication in PLOS Biology. On behalf of my colleagues and the Academic Editor, Roland Regoes, I'm pleased to say that we can in principle accept your manuscript for publication, provided you address any remaining formatting and reporting issues. These will be detailed in an email you should receive within 2-3 business days from our colleagues in the journal operations team; no action is required from you until then. Please note that we will not be able to formally accept your manuscript and schedule it for publication until you have completed any requested changes.

Please take a minute to log into Editorial Manager at http://www.editorialmanager.com/pbiology/, click the "Update My Information" link at the top of the page, and update your user information to ensure an efficient production process.

PRESS: We frequently collaborate with press offices. If your institution or institutions have a press office, please notify them about your upcoming paper at this point, to enable them to help maximise its impact. If the press office is planning to promote your findings, we would be grateful if they could coordinate with biologypress@plos.org. If you have previously opted in to the early version process, we ask that you notify us immediately of any press plans so that we may opt out on your behalf.

We also ask that you take this opportunity to read our Embargo Policy regarding the discussion, promotion and media coverage of work that is yet to be published by PLOS. As your manuscript is not yet published, it is bound by the conditions of our Embargo Policy. Please be aware that this policy is in place both to ensure that any press coverage of your article is fully substantiated and to provide a direct link between such coverage and the published work. For full details of our Embargo Policy, please visit http://www.plos.org/about/media-inquiries/embargo-policy/.

Thank you again for choosing PLOS Biology for publication and supporting Open Access publishing. We look forward to publishing your study.

Sincerely,

Roli

Roland G Roberts, PhD, PhD

Senior Editor

PLOS Biology

rroberts@plos.org
